# Genomic characterization of the human mitochondrial tumor suppressor gene 1 (*MTUS1*): 5' cloning and preliminary analysis of the multiple gene promoters

**DOI:** 10.1186/1756-0500-2-109

**Published:** 2009-06-19

**Authors:** Jinsheng Yu, Xiqiang Liu, Hui Ye, Xiaofeng Zhou

**Affiliations:** 1 Center for Molecular Biology of Oral Diseases, College of Dentistry, University of Illinois at Chicago, Chicago, IL, USA; 2 Guanghua School & Research Institute of Stomatology, Sun Yat-Sen University, Guangzhou, PR China

## Abstract

**Background:**

Mitochondrial tumor suppressor gene 1 (*MTUS1*) has been recently identified as a candidate tumor suppressor gene which resides in a genomic region (8p22) that shows frequent loss of heterozygosity (LOH) in several tumor types. It has been suggested that multiple gene promoters and alternative splicing lead to the expression of 5 known *MTUS1 *transcript variants.

**Findings:**

Here, we characterized the 5' untranslated regions of the different transcript variants. We also cloned and functionally tested the alternatively utilized gene promoters that contribute to the production of different *MTUS1 *transcript variants.

**Conclusion:**

Our results confirmed the early hypothesis that the transcript variants of *MTUS1 *gene are driven by multiple gene promoters.

## Introduction

The *MTUS1 *gene is located in a region (8p22) that shows frequent loss of heterozygosity (LOH) in several tumor types, including oral cancer [1]. Alternative exon utilization leads to the production of 5 known transcript variants (designated as variant 1 to 5) [2]. It has been suggested that the long form of transcript variants (variant 1, 2, and 3) are driven by a common gene promoter, while variant 4 and 5 are driven by 2 additional promoters [2]. Variant 5 was the first transcript variant to be cloned independently in 2 laboratories, as a gene that is transiently upregulated during initiation of cell differentiation and quiescence [3]. It represents an early component of the growth-inhibiting signaling cascade that interacts with angiotensin II AT2 receptor [4]. Evidence supporting the tumor suppressor function of other *MTUS1 *variants comes from the study on Xenopus *Icis *gene, a homolog of *MTUS1 *variants 1 and 2, which regulates microtubule growth and spindle formation prior to anaphase [5]. Here, we refined the genomic structure of the *MTUS1 *gene and functionally cloned the alternatively utilized gene promoters that control the production of these *MTUS1 *transcript variants. This will enhance our understanding on the regulation of this candidate tumor suppressor gene.

## Materials and methods

To characterize the 5' untranslated regions (5'-UTR) of the *MTUS1 *transcript variants, 5'-RACE assays were carried out using human brain reference mRNA (Ambion Inc) and a FirstChoice RLM-RACE kit from Ambion, with primers specific for various transcript variants (Additional file 1). The RACE products were PCR amplified, gel purified and then sequenced. The sequence results have been submitted to the GenBank (accession numbers: FJ458439, FJ458440, FJ458441, FJ472826, and FJ472827 for exon -1a, -1b, -1c, 5, and 8, respectively).

The gene promoter prediction was carried out using MatInspector Professional . The predicted transcription elements of the putative promoters were listed in Additional file 2. To assess the activities of potential gene promoters that control the productions of *MTUS1 *transcript variants, the following 4 fragments were PCR amplified using specific primers (Additional file 3) and Human Reference Genomic DNA (Promega): 1) a 2286 bp fragment (P1) located at the 5' flanking region of the *MTUS1 *gene; 2) a 773 bp fragment (P1') located at the 5' flanking region of exon 1; 3) a 529 bp fragment (P2) located at 5' flanking region of exon 5; and 4) a 733 bp fragment (P3) located at 5' flanking region of exon 8. The PCR products were then cloned into the KpnI/XhoI sites of pGL4.10 vector. After verification by DNA sequencing, the constructs were transiently transfected into cells using lipofectamine 2000 (Invitrogen). The pGL4.74 vector (Promega) was co-transfected as internal control for normalization of the transfection efficiency. After 48 hours, transfected cells were harvested with ice-cold phosphate-buffered saline, and dual luciferase assay were performed according to the manufacturer's protocol (Promega) using a Lumat LB 9507 Luminometer (Berthold Technologies).

## Results and Discussion

**Figure 1:**
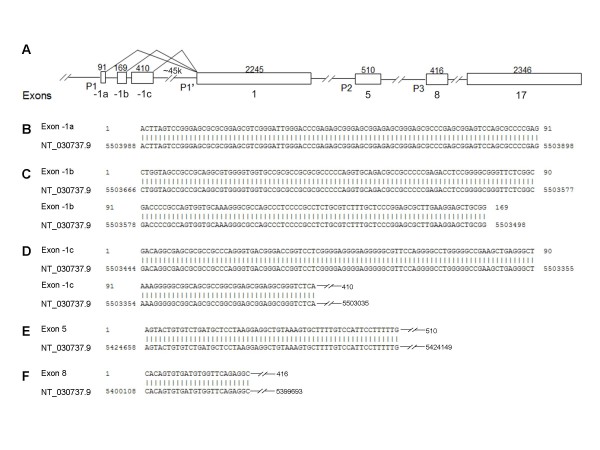
**Genomic characterization of the *MTUS1 *gene**. Schematic genomic organization of *MTUS1 *gene is presented in (**A**). Exons are indicated as boxes with corresponding sizes (bp) indicated above. Alternatively utilized gene promoters (P1, P1', P2, and P3) are indicated in the appropriated locations. 5'-RACE assays were carried out to characterize the 5' untranslated regions (5'-UTRs) corresponding to the long forms of transcription variants (variant 1, 2 and 3), and short forms of transcript variants (variant 4 and 5). The sequence alignments of the 5'-UTRs with human chromosome 8 reference sequence (NT_030737.9), which locate at exon -1a, -1b, -1c, 5 and 8 of the human *MTUS1 *gene are presented in (**B**) to (**F**), respectively. The corresponding GenBank accession numbers for exon -1a, -1b, -1c, 5, and 8 are FJ458439, FJ458440, FJ458441, FJ472826, and FJ472827, respectively.

To characterize the 5'-UTRs of *MTUS1 *transcription variants that are controlled by 3 different gene promoters, 5' RACE assays were performed as described in Materials and Methods section. The 5'RACE assay designed for the long forms of *MTUS1 *transcription variants (variant 1, 2 and 3) leads to the cloning of the 3 alternatively utilized exons of 91 bp, 169 bp, and 410 bp, respectively (Figure 1B–D). The 3' portion of the exon -1c (281 bp fragment) was previously reported in GenBank (NM_001001924). RT-PCR assays confirm the existence of these non-coding exons (data not shown). The 5'RACE assays designed for transcription variant 4 and 5 confirm the existence of these variants and also refined the 5' untranslated region for variant 5 (Figure 1E and 1F).

Based on sequence conservation and cap-analysis gene expression (CAGE) database, 3 gene promoters were predicted: P1, located at the 5'-flanking region of the gene; P2, located in intron region 5'-flanking exon 5; and P3, located in intron region 5'-flanking exon 8. These gene promoters were supported by sequence conservation (observed in 4, 9, and 11 of 11 orthologous loci analyzed, respectively) and CAGE database (supported by 29, 1 and 13 CAGE tages, respectively). The genomic locations of these putative promoters support the existence of multiple transcript variants of *MTUS1 *gene (Figure 1A). Interestingly, consensus sequences of relevant transcription elements (TATA-box and multiple CAAT sites) were also observed in a 500 bp fragment located in the intron 5'-flanking the exon 1 (named P1'), which may potentially contribute to the expression of the long transcription variants. We cloned these potential gene promoters and reporter gene assays were performed to evaluate the activities of these promoters using dual luciferase assay in 4 different cell lines, including a human lung fibroblast cell line (WI38), an immortalized normal oral keratinocyte (NOK-16B) and 2 oral tongue SCC cell lines (UM1 and UM2). As illustrated in Table 1, strong promoter activities were observed for P3 in all 4 cell lines tested. Promoter activities were also observed for P1 and P2 in UM1, UM2 and NOK16B, but not in WI38 cells. It is worth noting that transcript variant 4 (controlled by promoter P2) is a brain specific variant [2], which may explains the observed low activity of P2 promoter in our cell lines. No activity was found for negative control (empty pGL4.10 vector). These results confirmed the early hypothesis that the transcript variants of *MTUS1 *gene are driven by multiple gene promoters. Interestingly, P1', the putative promoter located in the intron region 5'-flanking exon 1 also exhibited strong promoter activity in all 4 cell lines tested, which suggests that the long form of transcript variants (variant 1, 2, and 3) may be regulated by both P1 and P1' promoters. It is possible that these 2 gene promoters may serve a cooperated control circuit *in vivo *to regulate the expression of this gene. This interesting phenomenon has been observed in a number of other genes, including gene for bradykinin B1 receptor [6], and proto-oncogene MDM2 [7]. Future studies will be needed to fully explore the potential interaction of P1 and P1' promoters.

**Table 1:** *MTUS1 *gene promoter activity measured by Dual Luciferase assay^1,2^

	UM1	UM2	NOK16B	WI38
P1	2.25 (0.42)	2.60 (0.20)	3.03 (0.61)	0.99 (0.08)
P1'	2.63 (0.73)	5.03 (0.74)	14.67 (0.21)	2.72 (0.01)
P2	1.29 (0.02)	1.34 (0.20)	1.52 (0.19)	1.01 (0.29)
P3	4.05 (0.57)	3.43 (1.33)	5.48 (2.57)	6.59 (1.01)

^1 ^Results presented are averages of at least 2 independent experiments of triplicate measurements. Standard deviations are presented in parentheses.

^2 ^Promoter activities are calculated as the ratios of the averages (Firefly/*Renilla*) from tested samples and averages (Firefly/*Renilla*) from negative controls.

In summary, our results refined the genomic structure of the *MTUS1 *gene and demonstrated the presence of multiple gene promoters. Characterization of this gene will now facilitate studies on the physiological and pathophysiological function and transcriptional regulation of the *MTUS1*.

## Competing interests

The authors declare that they have no competing interests.

## Authors' contributions

HY and XZ conceived the idea for the project. JY and XL performed the laboratory analyses. JY, HY and XZ drafted the manuscript. All authors read and approved the final manuscript.

## Supplementary Material

Additional file 1 **Table S1**. Gene-specific Primers used for the 5'-RACE assay. Click here for file

Additional file 2 **Table S2**. Predicted transcription elements in MTUS1 promoters. Click here for file

Additional file 3 **Table S3**. Primers used to clone promoter regions of the *MTUS1 *gene. Click here for file
