# Supplementary material for: Genomic characterization of the human mitochondrial tumor suppressor gene 1 (MTUS1): 5' cloning and preliminary analysis of the multiple gene promoters
Source: BMC Res Notes. 2009 Jun 19;2:109. doi: 10.1186/1756-0500-2-109 (PMC2706840; doi:10.1186/1756-0500-2-109)
Supplement: Additional file 1 — Table S1. Gene-specific Primers used for the 5'-RACE assay. [file 1756-0500-2-109-S1.doc]

**Supplementary Table 1**

**Gene-specific Primers used for the 5’-RACE assay**

| Exon | Outer Gene Specific Primer | Inner Gene Specific Primer (nested PCR) |
| --- | --- | --- |
| -1a | 5’-cca gtt cac act gct ggc tga a-3’ | 5’-agg gtg ggc aaa atg gtc tgt ctt-3’ |
| -1b |
| -1c |
| 5 | 5’-cca cga cca gca gtg tca at-3’ | 5’-aac tgc agc cat gtt cac tc-3’ |
| 8 | 5’-gca ggt ggc gag att tca ca-3’ | 5’-cca agt gtt tct ggc ttc cgt-3’ |

Note: Outer and Inner forward primers for 5’-RACE were supplied in the FirstChoice RLM-RACE kit (Ambion Inc.).
