# Supplementary material for: Genomic characterization of the human mitochondrial tumor suppressor gene 1 (MTUS1): 5' cloning and preliminary analysis of the multiple gene promoters
Source: BMC Res Notes. 2009 Jun 19;2:109. doi: 10.1186/1756-0500-2-109 (PMC2706840; doi:10.1186/1756-0500-2-109)
Supplement: Additional file 2 — Table S2. Predicted transcription elements in MTUS1 promoters. [file 1756-0500-2-109-S2.doc]

**Supplement Table 2: Predicted transcription elements in MTUS1 promoters**

| **Promoter** | **Family** | **Detailed Information** | **Matrix** | **Start pos.** | **End pos.** | **Strand** | **Core sim.** | **Matrix sim.** | **Sequence** |
| --- | --- | --- | --- | --- | --- | --- | --- | --- | --- |
| P1 | V$FKHD | Fork head domain factors | V$HNF3.01 | 13 | 29 | + | 1 | 1 | gtttagcAAACagcaag |
| P1 | V$FKHD | Fork head domain factors | V$FREAC2.01 | 36 | 52 | - | 1 | 0.889 | tttctcTAAAcacaaac |
| P1 | V$RORA | v-ERB and RAR-related orphan receptor alpha | V$RORA2.01 | 48 | 70 | + | 1 | 0.877 | agaaaagtaGGTCgagcctagtt |
| P1 | V$BRN5 | Brn-5 POU domain factors | V$BRN5.01 | 58 | 80 | + | 0.75 | 0.818 | gtcgagCCTAgtttatctccctg |
| P1 | V$HNF1 | Hepatic Nuclear Factor 1 | V$HMBOX.01 | 60 | 76 | + | 0.76 | 0.834 | cgagcctaGTTTatctc |
| P1 | V$CDXF | Vertebrate caudal related homeodomain protein | V$CDX2.02 | 62 | 80 | + | 1 | 0.864 | agcctagtTTATctccctg |
| P1 | V$FKHD | Fork head domain factors | V$HFH1.01 | 62 | 78 | - | 1 | 0.846 | gggagaTAAActaggct |
| P1 | V$GATA | GATA binding factors | V$GATA1.03 | 66 | 78 | - | 1 | 0.956 | gggaGATAaacta |
| P1 | V$NOLF | Neuron-specific-olfactory factor | V$OLF1.02 | 69 | 91 | + | 1 | 0.935 | tttatcTCCCtggggttctagac |
| P1 | V$HIFF | "Hypoxia inducible factor, bHLH/PAS protein family" | V$ARNT.01 | 94 | 110 | - | 1 | 0.906 | agatacaaCGTGgaaag |
| P1 | V$GATA | GATA binding factors | V$GATA2.02 | 101 | 113 | - | 1 | 0.916 | ttgaGATAcaacg |
| P1 | V$CART | Cart-1 (cartilage homeoprotein 1) | V$PHOX2.01 | 113 | 133 | + | 1 | 0.913 | agctcTAATagctggaaagct |
| P1 | V$NFAT | Nuclear factor of activated T-cells | V$NFAT5.01 | 123 | 141 | + | 1 | 0.872 | gctGGAAagctactttctt |
| P1 | V$MYT1 | MYT1 C2HC zinc finger protein | V$MYT1L.01 | 126 | 138 | + | 0.909 | 0.93 | ggaaAGCTacttt |
| P1 | V$HOMF | Homeodomain transcription factors | V$HHEX.01 | 138 | 156 | + | 1 | 0.959 | tctttaaccttTAATacac |
| P1 | V$KLFS | Krueppel like transcription factors | V$GKLF.02 | 138 | 156 | - | 1 | 0.974 | gtgtattAAAGgttaaaga |
| P1 | V$NBRE | "NGFI-B response elements, nur subfamily of nuclear receptors" | V$NBRE.01 | 138 | 152 | - | 1 | 0.863 | attaAAGGttaaaga |
| P1 | O$VTBP | Vertebrate TATA binding protein factor | O$ATATA.01 | 140 | 156 | - | 0.75 | 0.821 | gtgtattAAAGgttaaa |
| P1 | V$CART | Cart-1 (cartilage homeoprotein 1) | V$PHOX2.01 | 144 | 164 | + | 1 | 0.902 | accttTAATacacacttgcta |
| P1 | V$FAST | FAST-1 SMAD interacting proteins | V$FAST1.02 | 146 | 162 | - | 1 | 0.901 | gcaagTGTGtattaaag |
| P1 | V$HNF1 | Hepatic Nuclear Factor 1 | V$HMBOX.01 | 157 | 173 | + | 0.797 | 0.874 | acttgctaGTAAacttg |
| P1 | V$ABDB | Abdominal-B type homeodomain transcription factors | V$HOXB9.01 | 158 | 174 | + | 1 | 0.883 | cttgctagTAAActtgc |
| P1 | V$MYT1 | MYT1 C2HC zinc finger protein | V$MYT1.02 | 163 | 175 | - | 1 | 0.88 | agcAAGTttacta |
| P1 | V$BCL6 | POZ domain zinc finger expressed in B-Cells | V$BCL6.02 | 168 | 184 | + | 1 | 0.809 | aacttgcTAGAagcatg |
| P1 | V$BRN5 | Brn-5 POU domain factors | V$BRN5.02 | 171 | 193 | + | 0.75 | 0.774 | ttgctagaagcATGAagataata |
| P1 | V$BRN5 | Brn-5 POU domain factors | V$BRN5.02 | 181 | 203 | - | 1 | 0.777 | tcgcagtctgtATTAtcttcatg |
| P1 | V$GATA | GATA binding factors | V$GATA3.01 | 183 | 195 | + | 1 | 0.963 | tgaaGATAataca |
| P1 | V$PBXC | PBX1 - MEIS1 complexes | V$PBX1_MEIS1.01 | 195 | 211 | - | 1 | 0.763 | gggcTGATtcgcagtct |
| P1 | V$CAAT | CCAAT binding factors | V$NFY.02 | 197 | 211 | + | 0.75 | 0.833 | actgCGAAtcagccc |
| P1 | V$BCL6 | POZ domain zinc finger expressed in B-Cells | V$BCL6.02 | 207 | 223 | - | 1 | 0.807 | tgtctctTAGAagggct |
| P1 | V$PBXC | PBX1 - MEIS1 complexes | V$PBX1_MEIS1.01 | 215 | 231 | - | 1 | 0.765 | gtcgTGATtgtctctta |
| P1 | V$CAAT | CCAAT binding factors | V$NFY.03 | 217 | 231 | + | 0.75 | 0.838 | agagACAAtcacgac |
| P1 | V$GFI1 | Growth factor independence transcriptional repressor | V$GFI1B.01 | 220 | 234 | + | 1 | 0.862 | gacAATCacgactcc |
| P1 | O$TF2D | "General transcription factor IID, GTF2D" | O$INR_DPE.01 | 223 | 261 | - | 0.978 | 0.701 | attatcagggcggaagcttgcaaggaaggaGTCGtgatt |
| P1 | V$SP1F | GC-Box factors SP1/GC | V$BTEB3.01 | 225 | 239 | - | 1 | 0.956 | aggaaGGAGtcgtga |
| P1 | V$ETSF | Human and murine ETS1 factors | V$ELF2.01 | 226 | 246 | - | 1 | 0.905 | gcttgcaaGGAAggagtcgtg |
| P1 | V$BCL6 | POZ domain zinc finger expressed in B-Cells | V$BCL6.01 | 232 | 248 | + | 1 | 0.772 | tccTTCCttgcaagctt |
| P1 | V$ETSF | Human and murine ETS1 factors | V$SPI1_PU1.02 | 238 | 258 | - | 1 | 0.962 | atcagggcGGAAgcttgcaag |
| P1 | O$XCPE | "Activator-, mediator- and TBP-dependent core promoter element for RNA polymerase II transcription from TATA-less promoters" | O$XCPE1.01 | 244 | 254 | - | 1 | 0.905 | ggGCGGaagct |
| P1 | V$SP1F | GC-Box factors SP1/GC | V$SP1.02 | 244 | 258 | - | 1 | 0.852 | atcaGGGCggaagct |
| P1 | V$BRNF | Brn POU domain factors | V$TST1.01 | 248 | 266 | - | 1 | 0.927 | gcagaATTAtcagggcgga |
| P1 | V$HOMF | Homeodomain transcription factors | V$BARX2.01 | 251 | 269 | + | 1 | 0.954 | gccctgaTAATtctgcaac |
| P1 | V$GATA | GATA binding factors | V$GATA1.03 | 252 | 264 | + | 1 | 0.955 | ccctGATAattct |
| P1 | V$HEAT | Heat shock factors | V$HSF2.02 | 252 | 276 | - | 1 | 0.991 | tgggaaggttgcAGAAttatcaggg |
| P1 | V$PARF | PAR/bZIP family | V$HLF.01 | 256 | 272 | + | 0.836 | 0.857 | gataattctGCAAcctt |
| P1 | V$RBPF | RBPJ - kappa | V$RBPJK.02 | 266 | 280 | - | 1 | 0.964 | aaggTGGGaaggttg |
| P1 | V$PAX9 | PAX-9 binding sites | V$PAX9.01 | 279 | 299 | - | 1 | 0.814 | cgCACCgcggggcgtatagaa |
| P1 | V$NRSF | Neuron-restrictive silencer factor | V$NRSE.01 | 281 | 311 | + | 0.826 | 0.709 | ctatacgccccgCGGTgcgaggcccctcggc |
| P1 | V$CTCF | "CTCF and BORIS gene family, transcriptional regulators with 11 highly conserved zinc finger domains" | V$CTCF.04 | 303 | 329 | + | 1 | 0.936 | cccctcggcctgcaggTGGCcagaggc |
| P1 | V$CTCF | "CTCF and BORIS gene family, transcriptional regulators with 11 highly conserved zinc finger domains" | V$CTCF.04 | 303 | 329 | - | 0.857 | 0.895 | gcctctggccacctgcAGGCcgagggg |
| P1 | V$MYOD | Myoblast determining factors | V$E47.01 | 309 | 325 | + | 1 | 0.972 | ggcctGCAGgtggccag |
| P1 | V$RXRF | RXR heterodimer binding sites | V$RAR_RXR.01 | 311 | 335 | + | 0.769 | 0.79 | cctgcaggtggccagAGGCaagcag |
| P1 | V$NEUR | "NeuroD, Beta2, HLH domain" | V$NEUROD1.01 | 312 | 324 | - | 0.767 | 0.87 | tggcCACCtgcag |
| P1 | V$AP2F | Activator protein 2 | V$AP2.01 | 318 | 332 | + | 0.83 | 0.901 | gtgGCCAgaggcaag |
| P1 | V$AP2F | Activator protein 2 | V$AP2.01 | 318 | 332 | - | 1 | 0.924 | cttGCCTctggccac |
| P1 | V$BRAC | "Brachyury gene, mesoderm developmental factor" | V$BRACH.01 | 325 | 345 | + | 0.75 | 0.678 | gaggcaagcAGGAgtcagatc |
| P1 | V$NRSF | Neuron-restrictive silencer factor | V$NRSE.01 | 351 | 381 | + | 0.782 | 0.691 | cctaccgcggccCGGCctgggccctagcgct |
| P1 | V$PAX6 | PAX-4/PAX-6 paired domain binding sites | V$PAX6.02 | 361 | 379 | - | 1 | 0.871 | cgctagggcCCAGgccggg |
| P1 | V$ZFXY | Zfx and Zfy - transcription factors implicated in mammalian sex determination | V$ZFX.01 | 362 | 372 | + | 1 | 0.991 | ccGGCCtgggc |
| P1 | O$MTEN | Core promoter motif ten elements | O$DMTE.01 | 368 | 388 | - | 1 | 0.819 | cgcacccAGCGctagggccca |
| P1 | V$CP2F | CP2-erythrocyte Factor related to drosophila Elf1 | V$CP2.01 | 378 | 396 | + | 1 | 0.915 | cgCTGGgtgcggcggagcc |
| P1 | V$MZF1 | Myeloid zinc finger 1 factors | V$MZF1.01 | 407 | 417 | + | 1 | 0.991 | gcGGGGacgag |
| P1 | V$WHNF | Winged helix binding sites | V$WHN.01 | 427 | 437 | + | 1 | 0.952 | gggACGCgcgg |
| P1 | V$CP2F | CP2-erythrocyte Factor related to drosophila Elf1 | V$TCFCP2L1.01 | 430 | 448 | - | 1 | 0.87 | acCTGGctgcaccgcgcgt |
| P1 | V$HESF | Vertebrate homologues of enhancer of split complex | V$HES1.01 | 430 | 444 | + | 1 | 0.933 | acgcgcgGTGCagcc |
| P1 | V$HAND | Twist subfamily of class B bHLH transcription factors | V$TAL1_E2A.01 | 437 | 457 | + | 1 | 0.988 | gtgcagcCAGGtgcgccggcg |
| P1 | V$EBOX | E-box binding factors | V$MYCMAX.03 | 440 | 452 | + | 0.771 | 0.912 | cagccaGGTGcgc |
| P1 | V$ZFHX | Two-handed zinc finger homeodomain transcription factors | V$AREB6.03 | 441 | 453 | - | 1 | 0.96 | ggcgCACCtggct |
| P1 | V$EGRF | EGR/nerve growth factor induced protein C & related factors | V$EGR1.01 | 445 | 461 | + | 1 | 0.851 | aggtgcgccGGCGgcag |
| P1 | V$NRF1 | Nuclear respiratory factor 1 | V$NRF1.01 | 445 | 461 | - | 0.75 | 0.791 | ctgCCGCcggcgcacct |
| P1 | V$NRF1 | Nuclear respiratory factor 1 | V$NRF1.01 | 446 | 462 | + | 1 | 0.799 | ggtGCGCcggcggcagt |
| P1 | V$ETSF | Human and murine ETS1 factors | V$GABPB1.01 | 449 | 469 | + | 0.75 | 0.815 | gcgccggCGGCagtggcagcc |
| P1 | V$MYOD | Myoblast determining factors | V$MYF5.01 | 459 | 475 | + | 1 | 0.902 | cagtggCAGCcgctgag |
| P1 | V$WHNF | Winged helix binding sites | V$WHN.01 | 476 | 486 | - | 1 | 0.951 | gcgACGCggcg |
| P1 | V$HAND | Twist subfamily of class B bHLH transcription factors | V$SCX.01 | 479 | 499 | + | 1 | 0.931 | cgcgtcgcacgTGGCaacagg |
| P1 | V$HIFF | "Hypoxia inducible factor, bHLH/PAS protein family" | V$HIF1.01 | 480 | 496 | + | 1 | 0.982 | gcgtcgcACGTggcaac |
| P1 | V$HESF | Vertebrate homologues of enhancer of split complex | V$DEC2.01 | 481 | 495 | - | 1 | 0.983 | ttgccaCGTGcgacg |
| P1 | V$HIFF | "Hypoxia inducible factor, bHLH/PAS protein family" | V$ARNT.01 | 481 | 497 | - | 1 | 0.985 | tgttgccaCGTGcgacg |
| P1 | V$XBBF | X-box binding factors | V$RFX1.01 | 481 | 499 | + | 1 | 0.902 | cgtcgcacgtgGCAAcagg |
| P1 | V$EBOX | E-box binding factors | V$USF.01 | 482 | 494 | + | 1 | 0.993 | gtcgCACGtggca |
| P1 | V$HESF | Vertebrate homologues of enhancer of split complex | V$DEC2.01 | 482 | 496 | + | 1 | 0.975 | gtcgcaCGTGgcaac |
| P1 | V$EBOX | E-box binding factors | V$MYCMAX.03 | 483 | 495 | - | 0.982 | 0.994 | ttgccaCGTGcga |
| P1 | V$PERO | Peroxisome proliferator-activated receptor | V$PPAR_RXR.01 | 483 | 505 | + | 1 | 0.761 | tcgcacgtggcaacAGGTgcagc |
| P1 | V$MYOD | Myoblast determining factors | V$MYF5.01 | 487 | 503 | + | 0.963 | 0.903 | acgtggCAACaggtgca |
| P1 | V$HAND | Twist subfamily of class B bHLH transcription factors | V$TAL1_E2A.01 | 489 | 509 | + | 1 | 0.984 | gtggcaaCAGGtgcagcgctg |
| P1 | V$MYOD | Myoblast determining factors | V$MYOGENIN.01 | 490 | 506 | + | 1 | 0.975 | tggcaACAGgtgcagcg |
| P1 | V$ZFHX | Two-handed zinc finger homeodomain transcription factors | V$AREB6.03 | 493 | 505 | - | 1 | 0.981 | gctgCACCtgttg |
| P1 | V$BNCF | Basonuclein rDNA transcription factor (PolI) | V$BNC.01 | 498 | 516 | + | 1 | 0.92 | ggtgcagcgcTGTCccggg |
| P1 | V$NRF1 | Nuclear respiratory factor 1 | V$NRF1.01 | 510 | 526 | - | 1 | 0.783 | gcgGCGCcagcccggga |
| P1 | V$NRF1 | Nuclear respiratory factor 1 | V$NRF1.01 | 511 | 527 | + | 0.75 | 0.836 | cccGGGCtggcgccgcg |
| P1 | V$CTCF | "CTCF and BORIS gene family, transcriptional regulators with 11 highly conserved zinc finger domains" | V$CTCF.02 | 512 | 538 | - | 0.75 | 0.727 | gccccacgccccgcggcGCCAgcccgg |
| P1 | V$KLFS | Krueppel like transcription factors | V$KKLF.01 | 518 | 536 | + | 1 | 0.917 | tggcgccgcGGGGcgtggg |
| P1 | V$AHRR | AHR-arnt heterodimers and AHR-related factors | V$AHRARNT.03 | 520 | 544 | + | 1 | 0.952 | gcgccgcgggGCGTggggcgcgtcc |
| P1 | V$CTCF | "CTCF and BORIS gene family, transcriptional regulators with 11 highly conserved zinc finger domains" | V$CTCF.01 | 520 | 546 | + | 1 | 0.801 | gcgccgcggggcgtGGGGcgcgtccgc |
| P1 | V$PAX5 | PAX-5 B-cell-specific activator protein | V$PAX5.02 | 522 | 550 | + | 0.842 | 0.744 | gccgcggggcgtggGGCGcgtccgcactt |
| P1 | V$KLFS | Krueppel like transcription factors | V$GKLF.03 | 523 | 541 | + | 1 | 0.989 | ccgcggggcGTGGggcgcg |
| P1 | V$SP1F | GC-Box factors SP1/GC | V$TIEG.01 | 524 | 538 | + | 1 | 0.868 | cgcGGGGcgtggggc |
| P1 | V$NRF1 | Nuclear respiratory factor 1 | V$NRF1.01 | 533 | 549 | - | 0.75 | 0.849 | agtGCGGacgcgcccca |
| P1 | V$NRF1 | Nuclear respiratory factor 1 | V$NRF1.01 | 534 | 550 | + | 1 | 0.792 | gggGCGCgtccgcactt |
| P1 | V$NKXH | NKX homeodomain factors | V$NKX32.01 | 538 | 556 | - | 1 | 0.971 | cggactaAGTGcggacgcg |
| P1 | V$WHNF | Winged helix binding sites | V$WHN.01 | 565 | 575 | - | 1 | 0.966 | ccgACGCtccg |
| P1 | V$CLOX | CLOX and CLOX homology (CDP) factors | V$CDP.02 | 568 | 586 | - | 1 | 0.94 | gggtccCAATcccgacgct |
| P1 | V$GUCE | GTF2IRDI upstream control element | V$GTF3R4.01 | 573 | 583 | + | 1 | 0.97 | cggGATTggga |
| P1 | O$XCPE | "Activator-, mediator- and TBP-dependent core promoter element for RNA polymerase II transcription from TATA-less promoters" | O$XCPE1.01 | 589 | 599 | + | 1 | 0.924 | gaGCGGgagcg |
| P1 | V$SP1F | GC-Box factors SP1/GC | V$SP1.01 | 598 | 612 | + | 1 | 0.91 | cggaGGGCgggagcg |
| P1 | V$E2FF | E2F-myc activator/cell cycle regulator | V$E2F1_DP2.01 | 599 | 615 | + | 1 | 0.78 | ggagGGCGggagcgccc |
| P1 | V$EGRF | EGR/nerve growth factor induced protein C & related factors | V$WT1.01 | 600 | 616 | + | 0.953 | 0.928 | gagggCGGGagcgcccg |
| P1 | O$XCPE | "Activator-, mediator- and TBP-dependent core promoter element for RNA polymerase II transcription from TATA-less promoters" | O$XCPE1.01 | 602 | 612 | + | 1 | 0.978 | ggGCGGgagcg |
| P1 | V$DEAF | Homolog to deformed epidermal autoregulatory factor-1 from D. melanogaster | V$NUDR.01 | 602 | 620 | - | 1 | 0.788 | cgcTCGGgcgctcccgccc |
| P1 | V$NRF1 | Nuclear respiratory factor 1 | V$NRF1.01 | 606 | 622 | - | 0.75 | 0.826 | tccGCTCgggcgctccc |
| P1 | V$NRF1 | Nuclear respiratory factor 1 | V$NRF1.01 | 607 | 623 | + | 1 | 0.792 | ggaGCGCccgagcggag |
| P1 | O$MTEN | Core promoter motif ten elements | O$DMTE.01 | 610 | 630 | + | 1 | 0.82 | gcgcccgAGCGgagtccagcg |
| P1 | V$AHRR | AHR-arnt heterodimers and AHR-related factors | V$AHRARNT.02 | 639 | 663 | - | 0.75 | 0.784 | aacctgaatgACGTgcgaggccgta |
| P1 | V$CREB | cAMP-responsive element binding proteins | V$ATF6.02 | 641 | 661 | - | 1 | 0.977 | cctgaatGACGtgcgaggccg |
| P1 | V$HIFF | "Hypoxia inducible factor, bHLH/PAS protein family" | V$HIF1.01 | 643 | 659 | + | 1 | 0.901 | gcctcgcACGTcattca |
| P1 | V$CREB | cAMP-responsive element binding proteins | V$CREB.02 | 644 | 664 | - | 1 | 0.932 | gaacctgaaTGACgtgcgagg |
| P1 | V$HIFF | "Hypoxia inducible factor, bHLH/PAS protein family" | V$HRE.01 | 644 | 660 | - | 1 | 0.938 | ctgaatgaCGTGcgagg |
| P1 | V$PBXC | PBX1 - MEIS1 complexes | V$PBX1_MEIS1.03 | 647 | 663 | - | 1 | 0.784 | aacctgaaTGACgtgcg |
| P1 | V$NOLF | Neuron-specific-olfactory factor | V$OLF1.02 | 657 | 679 | + | 1 | 0.904 | tcaggtTCCCcgagggccgtccc |
| P1 | V$NFKB | Nuclear factor kappa B/c-rel | V$NFKAPPAB.01 | 669 | 681 | - | 1 | 0.892 | agGGGAcggccct |
| P1 | V$NOLF | Neuron-specific-olfactory factor | V$OLF1.02 | 669 | 691 | - | 1 | 0.898 | ccgccgTCCCaggggacggccct |
| P1 | V$NOLF | Neuron-specific-olfactory factor | V$OLF1.02 | 670 | 692 | + | 1 | 0.903 | gggccgTCCCctgggacggcggc |
| P1 | V$NR2F | Nuclear receptor subfamily 2 factors | V$HPF1.01 | 687 | 711 | - | 0.75 | 0.81 | ctgcgggagacCAAGgccagccgcc |
| P1 | V$EREF | Estrogen response elements | V$ERR.01 | 688 | 706 | - | 1 | 0.893 | ggagaccAAGGccagccgc |
| P1 | V$SF1F | Vertebrate steroidogenic factor | V$SF1.01 | 692 | 704 | - | 1 | 0.992 | agacCAAGgccag |
| P1 | V$PAX5 | PAX-5 B-cell-specific activator protein | V$PAX5.02 | 706 | 734 | - | 1 | 0.735 | gaaggctccactccAGCGgctcgctgcgg |
| P1 | O$MTEN | Core promoter motif ten elements | O$HMTE.01 | 708 | 728 | + | 1 | 0.911 | gcAGCGagccgctggagtgga |
| P1 | V$HEAT | Heat shock factors | V$HSF2.01 | 723 | 747 | - | 1 | 0.882 | ggcggctccttccGAAGgctccact |
| P1 | V$DEAF | Homolog to deformed epidermal autoregulatory factor-1 from D. melanogaster | V$NUDR.01 | 731 | 749 | - | 0.761 | 0.776 | gggGCGGctccttccgaag |
| P1 | V$ZBPF | Zinc binding protein factors | V$ZF9.01 | 736 | 758 | + | 1 | 0.944 | gaaggagCCGCcccgggttgggg |
| P1 | V$SP1F | GC-Box factors SP1/GC | V$SP1.01 | 738 | 752 | - | 1 | 0.887 | cccgGGGCggctcct |
| P1 | V$KLFS | Krueppel like transcription factors | V$KKLF.01 | 746 | 764 | + | 1 | 0.937 | ccccgggttGGGGcgggag |
| P1 | V$EGRF | EGR/nerve growth factor induced protein C & related factors | V$EGR1.02 | 748 | 764 | + | 1 | 0.901 | ccgggttgGGGCgggag |
| P1 | V$SP1F | GC-Box factors SP1/GC | V$SP1.01 | 752 | 766 | + | 1 | 0.951 | gttgGGGCgggaggc |
| P1 | V$EGRF | EGR/nerve growth factor induced protein C & related factors | V$WT1.01 | 754 | 770 | + | 0.953 | 0.922 | tggggCGGGaggcccga |
| P1 | O$XCPE | "Activator-, mediator- and TBP-dependent core promoter element for RNA polymerase II transcription from TATA-less promoters" | O$XCPE1.01 | 756 | 766 | + | 1 | 0.878 | ggGCGGgaggc |
| P1 | V$NFKB | Nuclear factor kappa B/c-rel | V$NFKAPPAB50.01 | 757 | 769 | + | 1 | 0.837 | ggcGGGAggcccg |
| P1 | V$DEAF | Homolog to deformed epidermal autoregulatory factor-1 from D. melanogaster | V$NUDR.01 | 770 | 788 | + | 1 | 0.861 | agtTCGGctatttacaagt |
| P1 | V$MEF2 | "MEF2, myocyte-specific enhancer binding factor" | V$SL1.01 | 770 | 792 | + | 1 | 0.866 | agttcggCTATttacaagtttcc |
| P1 | V$FKHD | Fork head domain factors | V$FREAC2.01 | 773 | 789 | - | 1 | 0.848 | aacttgTAAAtagccga |
| P1 | V$ABDB | Abdominal-B type homeodomain transcription factors | V$HOXA13.01 | 775 | 791 | - | 1 | 0.858 | gaaacttgTAAAtagcc |
| P1 | V$IRXF | Iroquois homeobox transcription factors | V$IRX4.01 | 779 | 791 | - | 0.777 | 0.841 | gaaaCTTGtaaat |
| P1 | V$HEAT | Heat shock factors | V$HSF1.01 | 780 | 804 | - | 0.952 | 0.905 | gcggggagttcgGGAAacttgtaaa |
| P1 | O$MTEN | Core promoter motif ten elements | O$HMTE.01 | 792 | 812 | - | 0.833 | 0.943 | ggAGCAgggcggggagttcgg |
| P1 | V$E2FF | E2F-myc activator/cell cycle regulator | V$E2F1_DP2.01 | 793 | 809 | - | 1 | 0.788 | gcagGGCGgggagttcg |
| P1 | V$KLFS | Krueppel like transcription factors | V$KKLF.01 | 793 | 811 | - | 1 | 0.959 | gagcagggcGGGGagttcg |
| P1 | V$MZF1 | Myeloid zinc finger 1 factors | V$MZF1.01 | 794 | 804 | - | 1 | 0.991 | gcGGGGagttc |
| P1 | V$MAZF | Myc associated zinc fingers | V$MAZ.01 | 796 | 808 | - | 0.866 | 0.913 | caggGCGGggagt |
| P1 | V$SP1F | GC-Box factors SP1/GC | V$SP1.01 | 796 | 810 | - | 1 | 0.934 | agcaGGGCggggagt |
| P1 | V$ZBPF | Zinc binding protein factors | V$ZBP89.01 | 804 | 826 | + | 1 | 0.953 | ccctgctccaCCCCctggcggcc |
| P1 | V$CTCF | "CTCF and BORIS gene family, transcriptional regulators with 11 highly conserved zinc finger domains" | V$CTCF.01 | 806 | 832 | - | 1 | 0.941 | ctccggggccgccaGGGGgtggagcag |
| P1 | V$CTCF | "CTCF and BORIS gene family, transcriptional regulators with 11 highly conserved zinc finger domains" | V$CTCF.04 | 806 | 832 | + | 0.841 | 0.887 | ctgctccaccccctggCGGCcccggag |
| P1 | V$KLFS | Krueppel like transcription factors | V$KLF6.01 | 806 | 824 | - | 1 | 0.93 | ccgccaGGGGgtggagcag |
| P1 | V$SP1F | GC-Box factors SP1/GC | V$GC.01 | 806 | 820 | - | 0.872 | 0.918 | cagggGGTGgagcag |
| P1 | V$AP2F | Activator protein 2 | V$AP2.02 | 823 | 837 | - | 1 | 0.938 | cccGCCTccggggcc |
| P1 | V$KLFS | Krueppel like transcription factors | V$KKLF.01 | 826 | 844 | + | 1 | 0.954 | cccggaggcGGGGaggcgg |
| P1 | V$SP1F | GC-Box factors SP1/GC | V$SP1.01 | 827 | 841 | + | 0.771 | 0.883 | ccggAGGCggggagg |
| P1 | V$MAZF | Myc associated zinc fingers | V$MAZ.01 | 829 | 841 | + | 0.866 | 0.91 | ggagGCGGggagg |
| P1 | V$PURA | Pur-alpha binds both single-stranded and douple-stranded DNA in a sequence-specific manner | V$PURALPHA.01 | 829 | 841 | + | 1 | 0.983 | ggAGGCggggagg |
| P1 | V$EGRF | EGR/nerve growth factor induced protein C & related factors | V$EGR1.02 | 831 | 847 | + | 0.842 | 0.903 | aggcggggAGGCgggga |
| P1 | V$MZF1 | Myeloid zinc finger 1 factors | V$MZF1.01 | 833 | 843 | + | 1 | 0.991 | gcGGGGaggcg |
| P1 | V$KLFS | Krueppel like transcription factors | V$KKLF.01 | 834 | 852 | + | 1 | 0.948 | cggggaggcGGGGaggccg |
| P1 | V$MAZF | Myc associated zinc fingers | V$MAZ.01 | 834 | 846 | + | 1 | 0.919 | cgggGAGGcgggg |
| P1 | V$SP1F | GC-Box factors SP1/GC | V$SP1.01 | 835 | 849 | + | 0.771 | 0.883 | ggggAGGCggggagg |
| P1 | V$MAZF | Myc associated zinc fingers | V$MAZ.01 | 837 | 849 | + | 0.866 | 0.91 | ggagGCGGggagg |
| P1 | V$PURA | Pur-alpha binds both single-stranded and douple-stranded DNA in a sequence-specific manner | V$PURALPHA.01 | 837 | 849 | + | 1 | 0.983 | ggAGGCggggagg |
| P1 | V$MZF1 | Myeloid zinc finger 1 factors | V$MZF1.01 | 841 | 851 | + | 1 | 0.991 | gcGGGGaggcc |
| P1 | V$PURA | Pur-alpha binds both single-stranded and douple-stranded DNA in a sequence-specific manner | V$PURALPHA.01 | 845 | 857 | + | 1 | 0.976 | ggAGGCcggggct |
| P1 | V$CP2F | CP2-erythrocyte Factor related to drosophila Elf1 | V$TCFCP2L1.01 | 856 | 874 | + | 0.926 | 0.897 | ctCCGGgtcccgcctggta |
| P1 | O$XCPE | "Activator-, mediator- and TBP-dependent core promoter element for RNA polymerase II transcription from TATA-less promoters" | O$XCPE1.01 | 860 | 870 | - | 1 | 0.877 | agGCGGgaccc |
| P1 | V$SP1F | GC-Box factors SP1/GC | V$SP2.01 | 860 | 874 | - | 1 | 0.871 | taccaggcgGGACcc |
| P1 | V$AP2F | Activator protein 2 | V$AP2.02 | 875 | 889 | - | 1 | 0.928 | cacGCCTgcggcggc |
| P1 | V$ZBPF | Zinc binding protein factors | V$ZF9.01 | 880 | 902 | - | 0.923 | 0.872 | cgcggcaCCACcccacgcctgcg |
| P1 | V$EGRF | EGR/nerve growth factor induced protein C & related factors | V$NGFIC.01 | 881 | 897 | + | 1 | 0.812 | gcagGCGTggggtggtg |
| P1 | V$SREB | Sterol regulatory element binding proteins | V$SREBP.02 | 884 | 898 | - | 0.75 | 0.884 | gcaCCACcccacgcc |
| P1 | V$GLIF | GLI zinc finger family | V$ZIC2.01 | 885 | 899 | - | 1 | 0.981 | ggcaccaCCCCacgc |
| P1 | V$SP1F | GC-Box factors SP1/GC | V$GC.01 | 886 | 900 | + | 0.872 | 0.892 | cgtggGGTGgtgccg |
| P1 | V$ZBPF | Zinc binding protein factors | V$ZF9.01 | 891 | 913 | + | 1 | 0.9 | ggtggtgCCGCgccgcgcgcccc |
| P1 | V$E2FF | E2F-myc activator/cell cycle regulator | V$E2F.03 | 892 | 908 | - | 1 | 0.9 | gcgcgGCGCggcaccac |
| P1 | O$XCPE | "Activator-, mediator- and TBP-dependent core promoter element for RNA polymerase II transcription from TATA-less promoters" | O$XCPE1.01 | 893 | 903 | - | 1 | 0.884 | gcGCGGcacca |
| P1 | O$TF2B | RNA polymerase II transcription factor II B | O$BRE.01 | 898 | 904 | + | 1 | 1 | ccgCGCC |
| P1 | V$EBOX | E-box binding factors | V$MYCMAX.03 | 900 | 912 | + | 1 | 0.943 | gcgccgCGCGccc |
| P1 | V$HESF | Vertebrate homologues of enhancer of split complex | V$HES1.01 | 900 | 914 | + | 0.944 | 0.958 | gcgccgcGCGCcccc |
| P1 | V$CTCF | "CTCF and BORIS gene family, transcriptional regulators with 11 highly conserved zinc finger domains" | V$CTCF.04 | 902 | 928 | - | 0.949 | 0.853 | cggcgtctgcacctggGGGCgcgcggc |
| P1 | V$HAND | Twist subfamily of class B bHLH transcription factors | V$TAL1_E2A.01 | 907 | 927 | + | 1 | 0.998 | gcgccccCAGGtgcagacgcc |
| P1 | V$HESF | Vertebrate homologues of enhancer of split complex | V$DEC2.01 | 909 | 923 | - | 0.87 | 0.963 | tctgcaCCTGggggc |
| P1 | V$ZFHX | Two-handed zinc finger homeodomain transcription factors | V$AREB6.03 | 911 | 923 | - | 1 | 0.981 | tctgCACCtgggg |
| P1 | V$AP1R | MAF and AP1 related factors | V$MAFA.01 | 915 | 935 | + | 0.904 | 0.925 | aggTGCAgacgccgcccccga |
| P1 | V$ZBPF | Zinc binding protein factors | V$ZF9.01 | 919 | 941 | + | 1 | 0.983 | gcagacgCCGCccccgagacctc |
| P1 | V$SP1F | GC-Box factors SP1/GC | V$SP1.02 | 921 | 935 | - | 1 | 0.919 | tcggGGGCggcgtct |
| P1 | V$ZBPF | Zinc binding protein factors | V$ZNF219.01 | 922 | 944 | + | 1 | 0.934 | gacgccgCCCCcgagacctccgg |
| P1 | V$EGRF | EGR/nerve growth factor induced protein C & related factors | V$EGR1.02 | 923 | 939 | - | 1 | 0.913 | ggtctcggGGGCggcgt |
| P1 | V$CTCF | "CTCF and BORIS gene family, transcriptional regulators with 11 highly conserved zinc finger domains" | V$CTCF.01 | 929 | 955 | + | 1 | 0.809 | cccccgagacctccGGGGcgggttctc |
| P1 | V$ETSF | Human and murine ETS1 factors | V$CETS1P54.01 | 930 | 950 | - | 1 | 0.926 | cccgccCCGGaggtctcgggg |
| P1 | V$ZBPF | Zinc binding protein factors | V$ZF9.01 | 934 | 956 | - | 1 | 0.935 | cgagaacCCGCcccggaggtctc |
| P1 | V$SP1F | GC-Box factors SP1/GC | V$SP1.01 | 940 | 954 | + | 1 | 0.97 | tccgGGGCgggttct |
| P1 | V$HEAT | Heat shock factors | V$HSF1.02 | 942 | 966 | - | 1 | 0.765 | gcggggtcgcCGAGaacccgccccg |
| P1 | O$XCPE | "Activator-, mediator- and TBP-dependent core promoter element for RNA polymerase II transcription from TATA-less promoters" | O$XCPE1.01 | 944 | 954 | + | 1 | 0.829 | ggGCGGgttct |
| P1 | V$XBBF | X-box binding factors | V$MIF1.01 | 946 | 964 | + | 0.75 | 0.797 | gcgggttctcgGCGAcccc |
| P1 | V$NR2F | Nuclear receptor subfamily 2 factors | V$HNF4.01 | 965 | 989 | + | 1 | 0.897 | gccagtggtgCAAAgggcgccagcc |
| P1 | V$LEFF | LEF1/TCF | V$LEF1.01 | 968 | 984 | + | 1 | 0.891 | agtggtgCAAAgggcgc |
| P1 | V$EGRF | EGR/nerve growth factor induced protein C & related factors | V$CKROX.01 | 982 | 998 | - | 1 | 0.887 | ggcgGGGAgggctggcg |
| P1 | V$SP1F | GC-Box factors SP1/GC | V$SP1.01 | 984 | 998 | - | 0.807 | 0.891 | ggcgGGGAgggctgg |
| P1 | V$E2FF | E2F-myc activator/cell cycle regulator | V$E2F1_DP1.01 | 986 | 1002 | - | 1 | 0.822 | cagaGGCGgggagggct |
| P1 | V$KLFS | Krueppel like transcription factors | V$KKLF.01 | 986 | 1004 | - | 1 | 0.966 | cgcagaggcGGGGagggct |
| P1 | V$MZF1 | Myeloid zinc finger 1 factors | V$MZF1.03 | 987 | 997 | - | 1 | 0.994 | gcGGGGagggc |
| P1 | V$MAZF | Myc associated zinc fingers | V$MAZ.01 | 989 | 1001 | - | 0.866 | 0.91 | agagGCGGggagg |
| P1 | V$PAX5 | PAX-5 B-cell-specific activator protein | V$PAX5.01 | 990 | 1018 | - | 0.904 | 0.806 | tccgggAGCAaagacgcagaggcggggag |
| P1 | O$MTEN | Core promoter motif ten elements | O$DMTE.01 | 1010 | 1030 | - | 1 | 0.785 | tccttcaAGCGctccgggagc |
| P1 | V$DEAF | Homolog to deformed epidermal autoregulatory factor-1 from D. melanogaster | V$NUDR.01 | 1031 | 1049 | + | 0.761 | 0.732 | gctGCGGgtaagtgcgcgc |
| P1 | V$NKXH | NKX homeodomain factors | V$NKX32.01 | 1034 | 1052 | + | 1 | 0.97 | gcgggtaAGTGcgcgctgg |
| P1 | V$CTCF | "CTCF and BORIS gene family, transcriptional regulators with 11 highly conserved zinc finger domains" | V$CTCF.02 | 1057 | 1083 | + | 0.75 | 0.709 | tgttcccgctgcccggcGGCCggggtg |
| P1 | V$CTCF | "CTCF and BORIS gene family, transcriptional regulators with 11 highly conserved zinc finger domains" | V$CTCF.01 | 1068 | 1094 | + | 0.789 | 0.842 | cccggcggccggggTGGGgaggggaca |
| P1 | V$ZBPF | Zinc binding protein factors | V$ZNF202.01 | 1069 | 1091 | - | 1 | 0.776 | cccctcCCCAccccggccgccgg |
| P1 | V$KLFS | Krueppel like transcription factors | V$BKLF.01 | 1074 | 1092 | + | 1 | 0.976 | ggccgGGGTggggagggga |
| P1 | V$SP1F | GC-Box factors SP1/GC | V$SP1.01 | 1075 | 1089 | + | 0.771 | 0.895 | gccgGGGTggggagg |
| P1 | V$MAZF | Myc associated zinc fingers | V$MAZ.01 | 1077 | 1089 | + | 0.8 | 0.911 | cgggGTGGggagg |
| P1 | V$EGRF | EGR/nerve growth factor induced protein C & related factors | V$CKROX.01 | 1080 | 1096 | + | 1 | 0.885 | ggtgGGGAggggacagg |
| P1 | V$SP1F | GC-Box factors SP1/GC | V$SP1.01 | 1080 | 1094 | + | 0.807 | 0.889 | ggtgGGGAggggaca |
| P1 | V$MZF1 | Myeloid zinc finger 1 factors | V$MZF1.03 | 1081 | 1091 | + | 1 | 1 | gtGGGGagggg |
| P1 | V$MAZF | Myc associated zinc fingers | V$MAZ.01 | 1082 | 1094 | + | 1 | 0.949 | tgggGAGGggaca |
| P1 | V$BNCF | Basonuclein rDNA transcription factor (PolI) | V$BNC.01 | 1086 | 1104 | - | 1 | 0.873 | cgcgctcgccTGTCccctc |
| P1 | V$MZF1 | Myeloid zinc finger 1 factors | V$MZF1.01 | 1086 | 1096 | + | 1 | 0.991 | gaGGGGacagg |
| P1 | O$MTEN | Core promoter motif ten elements | O$DMTE.01 | 1092 | 1112 | + | 1 | 0.811 | acaggcgAGCGcgccgcccag |
| P1 | V$NRF1 | Nuclear respiratory factor 1 | V$NRF1.01 | 1092 | 1108 | - | 1 | 0.782 | gcgGCGCgctcgcctgt |
| P1 | V$NRF1 | Nuclear respiratory factor 1 | V$NRF1.01 | 1093 | 1109 | + | 0.75 | 0.787 | cagGCGAgcgcgccgcc |
| P1 | O$MTEN | Core promoter motif ten elements | O$HMTE.01 | 1097 | 1117 | + | 1 | 0.909 | cgAGCGcgccgcccagggtga |
| P1 | V$SP1F | GC-Box factors SP1/GC | V$SP1.02 | 1100 | 1114 | - | 1 | 0.912 | ccctGGGCggcgcgc |
| P1 | V$KLFS | Krueppel like transcription factors | V$EKLF.01 | 1102 | 1120 | + | 1 | 0.925 | gcgccgcccaGGGTgacgg |
| P1 | V$CREB | cAMP-responsive element binding proteins | V$CREB.02 | 1106 | 1126 | + | 1 | 0.94 | cgcccagggTGACgggaccgg |
| P1 | V$CREB | cAMP-responsive element binding proteins | V$ATF2.01 | 1109 | 1129 | + | 1 | 0.89 | ccagggTGACgggaccggtcc |
| P1 | V$NOLF | Neuron-specific-olfactory factor | V$OLF1.02 | 1120 | 1142 | - | 1 | 0.894 | ctccccTCCCcgaggaccggtcc |
| P1 | V$NOLF | Neuron-specific-olfactory factor | V$OLF1.01 | 1121 | 1143 | + | 0.806 | 0.824 | gaccggTCCTcggggaggggagg |
| P1 | V$CTCF | "CTCF and BORIS gene family, transcriptional regulators with 11 highly conserved zinc finger domains" | V$CTCF.01 | 1128 | 1154 | + | 1 | 0.854 | cctcggggaggggaGGGGgcgttccag |
| P1 | V$KLFS | Krueppel like transcription factors | V$KKLF.01 | 1128 | 1146 | + | 1 | 0.982 | cctcggggaGGGGaggggg |
| P1 | V$ZBPF | Zinc binding protein factors | V$ZF9.01 | 1128 | 1150 | - | 0.82 | 0.888 | aacgcccCCTCccctccccgagg |
| P1 | V$EGRF | EGR/nerve growth factor induced protein C & related factors | V$CKROX.01 | 1129 | 1145 | + | 1 | 0.885 | ctcgGGGAggggagggg |
| P1 | V$SP1F | GC-Box factors SP1/GC | V$SP1.01 | 1129 | 1143 | + | 0.807 | 0.911 | ctcgGGGAggggagg |
| P1 | V$KLFS | Krueppel like transcription factors | V$KLF6.01 | 1131 | 1149 | + | 1 | 0.928 | cggggaGGGGagggggcgt |
| P1 | V$MAZF | Myc associated zinc fingers | V$MAZ.01 | 1131 | 1143 | + | 1 | 1 | cgggGAGGggagg |
| P1 | V$ZBPF | Zinc binding protein factors | V$ZNF219.01 | 1131 | 1153 | - | 1 | 0.989 | tggaacgCCCCctcccctccccg |
| P1 | V$EGRF | EGR/nerve growth factor induced protein C & related factors | V$CKROX.01 | 1134 | 1150 | + | 1 | 0.939 | ggagGGGAgggggcgtt |
| P1 | V$KLFS | Krueppel like transcription factors | V$KKLF.01 | 1134 | 1152 | + | 1 | 0.944 | ggaggggagGGGGcgttcc |
| P1 | V$PURA | Pur-alpha binds both single-stranded and douple-stranded DNA in a sequence-specific manner | V$PURALPHA.01 | 1134 | 1146 | + | 1 | 0.983 | ggAGGGgaggggg |
| P1 | V$SP1F | GC-Box factors SP1/GC | V$GC.01 | 1134 | 1148 | + | 0.876 | 0.911 | ggaggGGAGggggcg |
| P1 | V$MZF1 | Myeloid zinc finger 1 factors | V$MZF1.03 | 1135 | 1145 | + | 1 | 0.994 | gaGGGGagggg |
| P1 | V$EGRF | EGR/nerve growth factor induced protein C & related factors | V$EGR1.02 | 1136 | 1152 | + | 1 | 0.941 | aggggaggGGGCgttcc |
| P1 | V$KLFS | Krueppel like transcription factors | V$KLF6.01 | 1136 | 1154 | + | 1 | 0.942 | aggggaGGGGgcgttccag |
| P1 | V$MAZF | Myc associated zinc fingers | V$MAZR.01 | 1136 | 1148 | + | 1 | 0.914 | aggggaGGGGgcg |
| P1 | V$NFKB | Nuclear factor kappa B/c-rel | V$CREL.01 | 1141 | 1153 | + | 1 | 0.946 | agggggcgTTCCa |
| P1 | V$INSM | Insulinoma associated factors | V$INSM1.01 | 1149 | 1161 | + | 1 | 0.919 | ttccaGGGGcctg |
| P1 | V$AP2F | Activator protein 2 | V$AP2.01 | 1154 | 1168 | + | 1 | 0.924 | gggGCCTgggggccg |
| P1 | V$ZFXY | Zfx and Zfy - transcription factors implicated in mammalian sex determination | V$ZFX.01 | 1154 | 1164 | + | 1 | 0.996 | ggGGCCtgggg |
| P1 | V$ZBPF | Zinc binding protein factors | V$ZF9.01 | 1176 | 1198 | - | 1 | 0.943 | ggcgctgCCGCcccctttagccc |
| P1 | V$SP1F | GC-Box factors SP1/GC | V$SP1.01 | 1182 | 1196 | + | 1 | 0.887 | aaggGGGCggcagcg |
| P1 | V$NRF1 | Nuclear respiratory factor 1 | V$NRF1.01 | 1184 | 1200 | - | 1 | 0.786 | ccgGCGCtgccgccccc |
| P1 | O$XCPE | "Activator-, mediator- and TBP-dependent core promoter element for RNA polymerase II transcription from TATA-less promoters" | O$XCPE1.01 | 1186 | 1196 | + | 1 | 0.894 | ggGCGGcagcg |
| P1 | V$NRF1 | Nuclear respiratory factor 1 | V$NRF1.01 | 1190 | 1206 | - | 0.75 | 0.805 | gctCCGCcggcgctgcc |
| P1 | V$NRF1 | Nuclear respiratory factor 1 | V$NRF1.01 | 1191 | 1207 | + | 1 | 0.801 | gcaGCGCcggcggagcg |
| P1 | O$MTEN | Core promoter motif ten elements | O$DMTE.01 | 1197 | 1217 | + | 1 | 0.813 | ccggcggAGCGgaggcgggtc |
| P1 | V$EGRF | EGR/nerve growth factor induced protein C & related factors | V$EGR1.02 | 1201 | 1217 | + | 0.842 | 0.904 | cggagcggAGGCgggtc |
| P1 | V$SP1F | GC-Box factors SP1/GC | V$SP2.01 | 1205 | 1219 | + | 0.772 | 0.836 | gcggaggcgGGTCtc |
| P1 | V$CHRE | "Carbohydrate response elements, consist of two E box motifs separated by 5 bp" | V$CHREBP_MLX.01 | 1208 | 1224 | - | 1 | 0.858 | CACGtgagacccgcctc |
| P1 | V$CREB | cAMP-responsive element binding proteins | V$XBP1.01 | 1212 | 1232 | + | 1 | 0.901 | cgggtctcACGTgggccagcg |
| P1 | V$HIFF | "Hypoxia inducible factor, bHLH/PAS protein family" | V$HIF1.02 | 1213 | 1229 | + | 1 | 0.974 | gggtctcaCGTGggcca |
| P1 | V$HESF | Vertebrate homologues of enhancer of split complex | V$DEC2.01 | 1214 | 1228 | - | 1 | 0.994 | ggcccaCGTGagacc |
| P1 | V$HIFF | "Hypoxia inducible factor, bHLH/PAS protein family" | V$HIF1.02 | 1214 | 1230 | - | 1 | 0.964 | ctggcccaCGTGagacc |
| P1 | V$EBOX | E-box binding factors | V$USF.01 | 1215 | 1227 | + | 1 | 0.986 | gtctCACGtgggc |
| P1 | V$HESF | Vertebrate homologues of enhancer of split complex | V$DEC2.01 | 1215 | 1229 | + | 1 | 0.973 | gtctcaCGTGggcca |
| P1 | V$EBOX | E-box binding factors | V$NMYC.01 | 1216 | 1228 | - | 1 | 0.987 | ggcccaCGTGaga |
| P1 | V$PAX6 | PAX-4/PAX-6 paired domain binding sites | V$PAX6.02 | 1218 | 1236 | + | 1 | 0.879 | tcacgtgggCCAGcgcaga |
| P1 | V$NRF1 | Nuclear respiratory factor 1 | V$NRF1.01 | 1220 | 1236 | - | 1 | 0.789 | tctGCGCtggcccacgt |
| P1 | V$NRF1 | Nuclear respiratory factor 1 | V$NRF1.01 | 1221 | 1237 | + | 0.75 | 0.795 | cgtGGGCcagcgcagag |
| P1 | V$NRSF | Neuron-restrictive silencer factor | V$NRSF.03 | 1229 | 1259 | + | 0.776 | 0.679 | agcgCAGAgcctgcggaagggacggatgcgg |
| P1 | V$ETSF | Human and murine ETS1 factors | V$SPI1_PU1.02 | 1235 | 1255 | + | 1 | 0.964 | gagcctgcGGAAgggacggat |
| P1 | V$PAX3 | PAX-3 binding sites | V$PAX3.01 | 1262 | 1280 | + | 1 | 0.761 | cTCGTcgctgtcaccttga |
| P1 | V$TALE | TALE homeodomain class recognizing TG motifs | V$MEIS1.01 | 1263 | 1279 | + | 1 | 0.971 | tcgtcgcTGTCaccttg |
| P1 | V$HOXH | HOX - MEIS1 heterodimers | V$MEIS1B_HOXA9.01 | 1270 | 1284 | + | 0.75 | 0.783 | TGTCaccttgaaagt |
| P1 | V$NRSF | Neuron-restrictive silencer factor | V$NRSE.01 | 1271 | 1301 | - | 0.826 | 0.711 | agtcaagcccctCGGTcactttcaaggtgac |
| P1 | V$SF1F | Vertebrate steroidogenic factor | V$SF1.01 | 1271 | 1283 | - | 1 | 0.956 | ctttCAAGgtgac |
| P1 | V$DEAF | Homolog to deformed epidermal autoregulatory factor-1 from D. melanogaster | V$NUDR.01 | 1275 | 1293 | - | 1 | 0.801 | cccTCGGtcactttcaagg |
| P1 | V$RXRF | RXR heterodimer binding sites | V$VDR_RXR.05 | 1276 | 1300 | + | 0.761 | 0.798 | cttgAAAGtgaccgaggggcttgac |
| P1 | V$ZBPF | Zinc binding protein factors | V$ZF9.01 | 1309 | 1331 | + | 1 | 0.873 | ccttacgCCGCccacccgggccc |
| P1 | V$CTCF | "CTCF and BORIS gene family, transcriptional regulators with 11 highly conserved zinc finger domains" | V$CTCF.01 | 1310 | 1336 | - | 0.789 | 0.827 | ccgccgggcccgggTGGGcggcgtaag |
| P1 | V$EGRF | EGR/nerve growth factor induced protein C & related factors | V$WT1.01 | 1311 | 1327 | - | 1 | 0.937 | ccgggTGGGcggcgtaa |
| P1 | V$SP1F | GC-Box factors SP1/GC | V$SP1.02 | 1311 | 1325 | - | 1 | 0.899 | gggtGGGCggcgtaa |
| P1 | V$EGRF | EGR/nerve growth factor induced protein C & related factors | V$EGR2.01 | 1313 | 1329 | - | 0.766 | 0.851 | gcccGGGTgggcggcgt |
| P1 | V$ZBPF | Zinc binding protein factors | V$ZNF202.01 | 1313 | 1335 | + | 1 | 0.748 | acgccgCCCAcccgggcccggcg |
| P1 | V$HEAT | Heat shock factors | V$HSF1.01 | 1336 | 1360 | - | 1 | 0.869 | aggggccctgcgAGAAggctgggac |
| P1 | V$CTCF | "CTCF and BORIS gene family, transcriptional regulators with 11 highly conserved zinc finger domains" | V$CTCF.01 | 1415 | 1441 | - | 1 | 0.813 | gcgaggcgccacgaGGGGtctttggga |
| P1 | V$EBOX | E-box binding factors | V$NMYC.02 | 1423 | 1435 | + | 1 | 0.939 | acccctCGTGgcg |
| P1 | O$MTEN | Core promoter motif ten elements | O$HMTE.01 | 1424 | 1444 | - | 1 | 0.939 | gtAGCGaggcgccacgagggg |
| P1 | V$EBOX | E-box binding factors | V$ATF6.01 | 1424 | 1436 | - | 1 | 0.959 | gcgCCACgagggg |
| P1 | V$HEAT | Heat shock factors | V$HSF1.01 | 1437 | 1461 | - | 1 | 0.846 | caaacaagctgcAGAAagtagcgag |
| P1 | V$FKHD | Fork head domain factors | V$HNF3.01 | 1451 | 1467 | - | 1 | 0.995 | aaagtgcAAACaagctg |
| P1 | V$AHRR | AHR-arnt heterodimers and AHR-related factors | V$AHRARNT.03 | 1460 | 1484 | - | 1 | 0.953 | tttttctagaGCGTgaaaaagtgca |
| P1 | V$E2FF | E2F-myc activator/cell cycle regulator | V$E2F.02 | 1463 | 1479 | - | 1 | 0.909 | ctagagcgtGAAAaagt |
| P1 | V$HEAT | Heat shock factors | V$HSF2.02 | 1466 | 1490 | + | 1 | 0.995 | ttttcacgctctAGAAaaatctcat |
| P1 | V$PAX6 | PAX-4/PAX-6 paired domain binding sites | V$PAX6.03 | 1466 | 1484 | + | 1 | 0.784 | ttttcACGCtctagaaaaa |
| P1 | V$BCL6 | POZ domain zinc finger expressed in B-Cells | V$BCL6.01 | 1469 | 1485 | - | 0.756 | 0.771 | attTTTCtagagcgtga |
| P1 | V$EVI1 | EVI1-myleoid transforming protein | V$MEL1.02 | 1482 | 1498 | - | 1 | 0.992 | taattaaGATGagattt |
| P1' | M$TFAM | Mitochondral HMG-box transcription factor | M$TFAM.01 | 16 | 28 | - | 1 | 0.983 | aatagttTTTTct |
| P1' | V$HAND | Twist subfamily of class B bHLH transcription factors | V$TH1E47.01 | 21 | 41 | + | 1 | 0.933 | aaactattgCCAGatttacct |
| P1' | V$HOXH | HOX - MEIS1 heterodimers | V$MEIS1B_HOXA9.01 | 28 | 42 | + | 0.75 | 0.811 | TGCCagatttacctc |
| P1' | V$ABDB | Abdominal-B type homeodomain transcription factors | V$HOXB9.01 | 30 | 46 | - | 1 | 0.895 | ggaggaggTAAAtctgg |
| P1' | V$EVI1 | EVI1-myleoid transforming protein | V$EVI1.04 | 36 | 52 | - | 0.75 | 0.743 | aagagaggaggaGGTAa |
| P1' | V$P53F | p53 tumor suppressor | V$P53.03 | 41 | 63 | + | 0.921 | 0.942 | tcctcctctctttgaCAAGtccc |
| P1' | V$LEFF | LEF1/TCF | V$LEF1.02 | 45 | 61 | - | 1 | 0.947 | gacttgtCAAAgagagg |
| P1' | V$TALE | TALE homeodomain class recognizing TG motifs | V$TGIF.01 | 48 | 64 | - | 1 | 1 | agggacttGTCAaagag |
| P1' | V$NFKB | Nuclear factor kappa B/c-rel | V$NFKAPPAB.02 | 53 | 65 | - | 1 | 0.842 | caGGGActtgtca |
| P1' | V$ZBPF | Zinc binding protein factors | V$ZNF219.01 | 60 | 82 | - | 1 | 0.949 | tgcctctCCCCcactcccaggga |
| P1' | V$KLFS | Krueppel like transcription factors | V$KKLF.01 | 63 | 81 | + | 1 | 0.938 | ctgggagtgGGGGagaggc |
| P1' | V$ZBPF | Zinc binding protein factors | V$ZBP89.01 | 63 | 85 | - | 1 | 0.959 | atatgcctctCCCCcactcccag |
| P1' | V$EGRF | EGR/nerve growth factor induced protein C & related factors | V$WT1.01 | 65 | 81 | + | 1 | 0.982 | gggagTGGGggagaggc |
| P1' | V$GATA | GATA binding factors | V$GATA.01 | 78 | 90 | - | 1 | 0.943 | aactGATAtgcct |
| P1' | V$OCT1 | Octamer binding protein | V$OCT1.05 | 79 | 95 | + | 0.85 | 0.915 | ggCATAtcagttataaa |
| P1' | V$MYBL | Cellular and viral myb-like transcriptional regulators | V$VMYB.01 | 81 | 93 | - | 0.865 | 0.899 | tatAACTgatatg |
| P1' | V$NRSF | Neuron-restrictive silencer factor | V$NRSF.03 | 82 | 112 | + | 0.764 | 0.678 | atatCAGTtataaagtcagggacagggttaa |
| P1' | V$ABDB | Abdominal-B type homeodomain transcription factors | V$HOXC9.01 | 84 | 100 | + | 0.884 | 0.841 | atcagttaTAAAgtcag |
| P1' | V$CDXF | Vertebrate caudal related homeodomain protein | V$CDX2.02 | 84 | 102 | - | 1 | 0.87 | ccctgactTTATaactgat |
| P1' | O$VTBP | Vertebrate TATA binding protein factor | O$VTATA.01 | 87 | 103 | + | 1 | 0.913 | agttaTAAAgtcaggga |
| P1' | V$KLFS | Krueppel like transcription factors | V$GKLF.01 | 90 | 108 | + | 1 | 0.872 | tataaagtcAGGGacaggg |
| P1' | V$CSEN | "Calsenilin, presenilin binding protein, EF hand transcription factor" | V$DREAM.01 | 94 | 104 | + | 1 | 0.992 | aaGTCAgggac |
| P1' | V$HNF1 | Hepatic Nuclear Factor 1 | V$HNF1.04 | 100 | 116 | + | 1 | 0.86 | gggacaggGTTAacgag |
| P1' | V$ABDB | Abdominal-B type homeodomain transcription factors | V$HOXA13.01 | 104 | 120 | - | 0.778 | 0.853 | attgctcgTTAAccctg |
| P1' | V$CREB | cAMP-responsive element binding proteins | V$TAXCREB.01 | 104 | 124 | + | 0.784 | 0.838 | cagggtTAACgagcaatgacc |
| P1' | V$HNF1 | Hepatic Nuclear Factor 1 | V$TCF2.01 | 105 | 121 | - | 1 | 0.904 | cattgctcGTTAaccct |
| P1' | V$MYBL | Cellular and viral myb-like transcriptional regulators | V$VMYB.04 | 108 | 120 | + | 1 | 0.897 | gttAACGagcaat |
| P1' | V$EVI1 | EVI1-myleoid transforming protein | V$MEL1.03 | 120 | 136 | - | 1 | 0.961 | aatggaaGATGgggtca |
| P1' | V$YY1F | Activator/repressor binding to transcription initiation site | V$YY1.02 | 120 | 138 | + | 1 | 0.947 | tgaccCCATcttccatttt |
| P1' | V$ETSF | Human and murine ETS1 factors | V$SPIB.01 | 121 | 141 | - | 1 | 0.913 | ggcaaaatGGAAgatggggtc |
| P1' | V$HAND | Twist subfamily of class B bHLH transcription factors | V$PARAXIS.01 | 135 | 155 | - | 0.882 | 0.877 | ggaatAGCAaatggggcaaaa |
| P1' | V$NEUR | "NeuroD, Beta2, HLH domain" | V$NEUROG.01 | 139 | 151 | + | 1 | 0.932 | gccCCATttgcta |
| P1' | V$STAT | Signal transducer and activator of transcription | V$STAT.01 | 146 | 164 | - | 1 | 0.904 | taatttgagGGAAtagcaa |
| P1' | V$IKRS | Ikaros zinc finger family | V$IK1.01 | 148 | 160 | - | 1 | 0.932 | ttgaGGGAatagc |
| P1' | V$LHXF | Lim homeodomain factors | V$LHX3.01 | 151 | 173 | - | 1 | 0.815 | atacttttTTAAtttgagggaat |
| P1' | V$HBOX | Homeobox transcription factors | V$GSH2.01 | 152 | 170 | - | 1 | 0.963 | ctttttTAATttgagggaa |
| P1' | V$HOMF | Homeodomain transcription factors | V$MSX.01 | 153 | 171 | - | 1 | 0.993 | actttttTAATttgaggga |
| P1' | V$CART | Cart-1 (cartilage homeoprotein 1) | V$ALX4.01 | 154 | 174 | + | 1 | 0.835 | ccctcaAATTaaaaaagtatc |
| P1' | V$HOXF | "Paralog hox genes 1-8 from the four hox clusters A, B, C, D" | V$HOXC5.01 | 154 | 172 | + | 1 | 0.823 | ccctcaAATTaaaaaagta |
| P1' | V$LHXF | Lim homeodomain factors | V$LHX3.02 | 155 | 177 | - | 1 | 0.864 | caagatactttttTAATttgagg |
| P1' | V$NKX6 | NK6 homeobox transcription factors | V$NKX61.01 | 155 | 169 | - | 1 | 0.92 | ttttTTAAtttgagg |
| P1' | V$BRNF | Brn POU domain factors | V$TST1.02 | 156 | 174 | + | 1 | 0.79 | ctcaaaTTAAaaaagtatc |
| P1' | V$RBIT | Regulator of B-Cell IgH transcription | V$BRIGHT.01 | 156 | 168 | + | 1 | 0.939 | ctcaaATTAaaaa |
| P1' | V$HOMF | Homeodomain transcription factors | V$HHEX.01 | 157 | 175 | - | 1 | 0.974 | agatactttttTAATttga |
| P1' | V$BRNF | Brn POU domain factors | V$BRN2.01 | 158 | 176 | + | 0.933 | 0.907 | caAATTaaaaaagtatctt |
| P1' | V$CLOX | CLOX and CLOX homology (CDP) factors | V$CLOX.01 | 179 | 197 | - | 0.806 | 0.814 | ggcaATCTatcaccgcttt |
| P1' | V$HOXC | HOX - PBX complexes | V$HOX_PBX.01 | 182 | 198 | + | 1 | 0.89 | gcggTGATagattgcct |
| P1' | V$CLOX | CLOX and CLOX homology (CDP) factors | V$CDPCR3HD.01 | 184 | 202 | + | 0.885 | 0.943 | ggtgataGATTgcctgatt |
| P1' | V$BRN5 | Brn-5 POU domain factors | V$BRN5.04 | 189 | 211 | + | 1 | 0.887 | tagattgcctgATTAtatctcaa |
| P1' | O$VTBP | Vertebrate TATA binding protein factor | O$LTATA.01 | 192 | 208 | - | 1 | 0.858 | agaTATAatcaggcaat |
| P1' | V$CLOX | CLOX and CLOX homology (CDP) factors | V$CPHX.01 | 192 | 210 | + | 1 | 0.847 | attgccTGATtatatctca |
| P1' | V$HNF6 | Onecut homeodomain factor HNF6 | V$OC2.01 | 192 | 208 | - | 1 | 0.852 | agatatAATCaggcaat |
| P1' | V$LHXF | Lim homeodomain factors | V$LHX3.02 | 194 | 216 | - | 1 | 0.857 | ggtaattgagataTAATcaggca |
| P1' | V$CART | Cart-1 (cartilage homeoprotein 1) | V$S8.01 | 199 | 219 | - | 1 | 1 | gtgggTAATtgagatataatc |
| P1' | V$GATA | GATA binding factors | V$GATA2.02 | 199 | 211 | - | 1 | 0.935 | ttgaGATAtaatc |
| P1' | V$DLXF | Distal-less homeodomain transcription factors | V$DLX5.01 | 200 | 218 | + | 1 | 0.943 | attatatctcAATTaccca |
| P1' | V$HOXF | "Paralog hox genes 1-8 from the four hox clusters A, B, C, D" | V$HOXC6.01 | 201 | 219 | - | 1 | 0.854 | gtgggtAATTgagatataa |
| P1' | V$HBOX | Homeobox transcription factors | V$VAX1.01 | 202 | 220 | - | 1 | 0.858 | tgtgggtAATTgagatata |
| P1' | V$HOMF | Homeodomain transcription factors | V$BSX.01 | 202 | 220 | + | 1 | 0.952 | tatatctcAATTacccaca |
| P1' | V$OCT1 | Octamer binding protein | V$OCT1.06 | 202 | 218 | + | 1 | 0.839 | tatatctcAATTaccca |
| P1' | V$HBOX | Homeobox transcription factors | V$MEOX1.01 | 203 | 221 | + | 1 | 0.849 | atatctcAATTacccacat |
| P1' | V$HOMF | Homeodomain transcription factors | V$BSX.01 | 203 | 221 | - | 1 | 0.959 | atgtgggtAATTgagatat |
| P1' | V$HOXF | "Paralog hox genes 1-8 from the four hox clusters A, B, C, D" | V$HOXC8.01 | 204 | 222 | + | 1 | 0.864 | tatctcaATTAcccacatc |
| P1' | V$DLXF | Distal-less homeodomain transcription factors | V$DLX1.01 | 205 | 223 | - | 1 | 0.928 | tgatgtgggtAATTgagat |
| P1' | V$BRNF | Brn POU domain factors | V$BRN4.01 | 207 | 225 | - | 1 | 0.909 | actgatgtgggTAATtgag |
| P1' | V$NR2F | Nuclear receptor subfamily 2 factors | V$HNF4.02 | 228 | 252 | + | 0.75 | 0.787 | ttgtggggtctAAATgtgaacataa |
| P1' | V$FKHD | Fork head domain factors | V$XFD3.01 | 238 | 254 | + | 1 | 0.876 | taaatgtgAACAtaaag |
| P1' | V$GREF | Glucocorticoid responsive and related elements | V$ARE.02 | 242 | 260 | - | 1 | 0.903 | caatcactttatGTTCaca |
| P1' | V$GREF | Glucocorticoid responsive and related elements | V$GRE.02 | 242 | 260 | + | 1 | 0.836 | tgtGAACataaagtgattg |
| P1' | V$NKXH | NKX homeodomain factors | V$NKX26.01 | 246 | 264 | + | 1 | 0.837 | aacataaAGTGattgaaac |
| P1' | V$CLOX | CLOX and CLOX homology (CDP) factors | V$CDP.02 | 248 | 266 | - | 1 | 0.943 | tagtttCAATcactttatg |
| P1' | V$HOXC | HOX - PBX complexes | V$PBX1.01 | 251 | 267 | + | 1 | 0.827 | aaagtGATTgaaactaa |
| P1' | V$PBXC | PBX1 - MEIS1 complexes | V$PBX1_MEIS1.02 | 251 | 267 | + | 1 | 0.826 | aaagTGATtgaaactaa |
| P1' | V$PDX1 | Pancreatic and intestinal homeodomain transcription factor | V$PDX1.01 | 256 | 274 | + | 1 | 0.812 | gattgaaacTAATcactta |
| P1' | V$HBOX | Homeobox transcription factors | V$EVX1.01 | 258 | 276 | - | 1 | 0.846 | actaagtgATTAgtttcaa |
| P1' | V$HBOX | Homeobox transcription factors | V$EVX1.01 | 259 | 277 | + | 0.775 | 0.845 | tgaaactaATCActtagtg |
| P1' | V$BCDF | Bicoid-like homeodomain transcription factors | V$CRX.01 | 260 | 276 | + | 1 | 0.947 | gaaacTAATcacttagt |
| P1' | V$CAAT | CCAAT binding factors | V$NFY.03 | 260 | 274 | + | 0.75 | 0.819 | gaaaCTAAtcactta |
| P1' | V$HOXF | "Paralog hox genes 1-8 from the four hox clusters A, B, C, D" | V$HOXB3.01 | 260 | 278 | + | 1 | 0.84 | gaaacTAATcacttagtga |
| P1' | V$LHXF | Lim homeodomain factors | V$ISL2.01 | 260 | 282 | + | 0.881 | 0.879 | gaaactaatcaCTTAgtgattga |
| P1' | V$OCT1 | Octamer binding protein | V$OCT1.03 | 260 | 276 | - | 1 | 0.855 | actaagtgATTAgtttc |
| P1' | V$NKXH | NKX homeodomain factors | V$NKX32.01 | 261 | 279 | - | 1 | 0.962 | atcactaAGTGattagttt |
| P1' | V$PDX1 | Pancreatic and intestinal homeodomain transcription factor | V$IPF1.01 | 261 | 279 | - | 0.764 | 0.848 | atcactaagTGATtagttt |
| P1' | V$BRN5 | Brn-5 POU domain factors | V$BRN5.03 | 264 | 286 | + | 1 | 0.789 | cTAATcacttagtgattgaaacc |
| P1' | V$CLOX | CLOX and CLOX homology (CDP) factors | V$CDP.02 | 269 | 287 | - | 1 | 0.943 | tggtttCAATcactaagtg |
| P1' | V$SORY | SOX/SRY-sex/testis determinig and related HMG box factors | V$HBP1.01 | 270 | 286 | + | 0.8 | 0.876 | acttagtgATTGaaacc |
| P1' | V$HOXC | HOX - PBX complexes | V$PBX1.01 | 272 | 288 | + | 1 | 0.823 | ttagtGATTgaaaccaa |
| P1' | V$PBXC | PBX1 - MEIS1 complexes | V$PBX1_MEIS1.02 | 272 | 288 | + | 1 | 0.832 | ttagTGATtgaaaccaa |
| P1' | V$CTCF | "CTCF and BORIS gene family, transcriptional regulators with 11 highly conserved zinc finger domains" | V$CTCF.04 | 277 | 303 | + | 1 | 0.852 | gattgaaaccaaggggTGGCctgttaa |
| P1' | V$SIXF | Sine oculis (SIX) homeodomain factors | V$SIX4.01 | 277 | 291 | - | 0.785 | 0.884 | cccttgGTTTcaatc |
| P1' | V$ZBPF | Zinc binding protein factors | V$ZF9.01 | 280 | 302 | - | 0.923 | 0.888 | taacaggCCACcccttggtttca |
| P1' | V$HOMF | Homeodomain transcription factors | V$HMX3.02 | 290 | 308 | + | 1 | 0.924 | gggtggcctgTTAAttttt |
| P1' | V$BRNF | Brn POU domain factors | V$TST1.01 | 291 | 309 | - | 1 | 0.917 | gaaaaATTAacaggccacc |
| P1' | V$MYBL | Cellular and viral myb-like transcriptional regulators | V$CMYB.02 | 292 | 304 | - | 1 | 0.961 | atTAACaggccac |
| P1' | V$SORY | SOX/SRY-sex/testis determinig and related HMG box factors | V$HMGA.01 | 292 | 308 | - | 1 | 0.886 | aaaAATTaacaggccac |
| P1' | V$FAST | FAST-1 SMAD interacting proteins | V$FAST1.01 | 293 | 309 | + | 0.85 | 0.823 | tggcctgttAATTtttc |
| P1' | V$BRNF | Brn POU domain factors | V$BRN3.02 | 294 | 312 | + | 1 | 0.9 | ggcctgtTAATttttctta |
| P1' | V$HOMF | Homeodomain transcription factors | V$BARHL2.01 | 294 | 312 | + | 1 | 0.831 | ggcctgtTAATttttctta |
| P1' | V$NKX6 | NK6 homeobox transcription factors | V$NKX61.01 | 296 | 310 | + | 1 | 0.916 | cctgTTAAtttttct |
| P1' | V$NKXH | NKX homeodomain factors | V$NKX25.02 | 296 | 314 | + | 1 | 0.884 | cctgtTAATttttcttata |
| P1' | V$HNF1 | Hepatic Nuclear Factor 1 | V$HNF1.01 | 298 | 314 | + | 1 | 0.907 | tGTTAatttttcttata |
| P1' | O$VTBP | Vertebrate TATA binding protein factor | O$LTATA.01 | 301 | 317 | - | 1 | 0.823 | aacTATAagaaaaatta |
| P1' | V$HNF1 | Hepatic Nuclear Factor 1 | V$HMBOX.01 | 307 | 323 | + | 1 | 0.837 | ttcttataGTTActtga |
| P1' | V$CREB | cAMP-responsive element binding proteins | V$E4BP4.01 | 309 | 329 | - | 1 | 0.819 | tttaactcaaGTAActataag |
| P1' | V$PARF | PAR/bZIP family | V$VBP.01 | 312 | 328 | - | 1 | 0.89 | ttaactcaaGTAActat |
| P1' | V$HNF1 | Hepatic Nuclear Factor 1 | V$HNF1.04 | 316 | 332 | + | 1 | 0.904 | ttacttgaGTTAaacat |
| P1' | V$FKHD | Fork head domain factors | V$HFH1.01 | 320 | 336 | + | 1 | 0.903 | ttgagtTAAAcatttta |
| P1' | W$RPOA | Retroviral PolyA signal | W$POLYA.01 | 321 | 341 | - | 1 | 0.796 | agaaaTAAAatgtttaactca |
| P1' | V$HNF1 | Hepatic Nuclear Factor 1 | V$HNF1.01 | 323 | 339 | + | 1 | 0.801 | aGTTAaacattttattt |
| P1' | V$HOMF | Homeodomain transcription factors | V$HMX2.02 | 324 | 342 | - | 0.75 | 0.823 | aagaaataAAATgtttaac |
| P1' | V$BRNF | Brn POU domain factors | V$TST1.01 | 325 | 343 | - | 0.9 | 0.941 | gaagaAATAaaatgtttaa |
| P1' | V$CDXF | Vertebrate caudal related homeodomain protein | V$CDX2.02 | 326 | 344 | + | 1 | 0.908 | taaacattTTATttcttcc |
| P1' | V$FKHD | Fork head domain factors | V$FHXB.01 | 327 | 343 | - | 1 | 0.831 | gaagaaATAAaatgttt |
| P1' | V$ABDB | Abdominal-B type homeodomain transcription factors | V$HOXA10.01 | 328 | 344 | - | 1 | 0.928 | ggaagaaaTAAAatgtt |
| P1' | V$OCT1 | Octamer binding protein | V$OCT1.05 | 328 | 344 | + | 0.9 | 0.932 | aaCATTttatttcttcc |
| P1' | V$STAT | Signal transducer and activator of transcription | V$STAT6.01 | 336 | 354 | - | 1 | 0.923 | gttgTTCCctggaagaaat |
| P1' | V$STAT | Signal transducer and activator of transcription | V$STAT3.02 | 337 | 355 | + | 1 | 0.969 | tttcTTCCagggaacaaca |
| P1' | V$IKRS | Ikaros zinc finger family | V$IK3.01 | 342 | 354 | + | 1 | 0.884 | tccagGGAAcaac |
| P1' | V$BPTF | Bromodomain and PHD domain transcription factors | V$FAC1.01 | 347 | 357 | + | 1 | 0.976 | ggaacAACAaa |
| P1' | V$HOXC | HOX - PBX complexes | V$PBX_HOXA9.01 | 348 | 364 | - | 1 | 0.845 | taaaTGATttgttgttc |
| P1' | V$CAAT | CCAAT binding factors | V$NFY.03 | 350 | 364 | + | 0.75 | 0.811 | acaaCAAAtcattta |
| P1' | V$LHXF | Lim homeodomain factors | V$ISL2.01 | 350 | 372 | + | 0.804 | 0.892 | acaacaaatcaTTTAatcttcag |
| P1' | V$GFI1 | Growth factor independence transcriptional repressor | V$GFI1B.01 | 353 | 367 | + | 1 | 0.863 | acaAATCatttaatc |
| P1' | V$HOMF | Homeodomain transcription factors | V$BARHL2.01 | 353 | 371 | - | 0.762 | 0.859 | tgaagatTAAAtgatttgt |
| P1' | V$NKX6 | NK6 homeobox transcription factors | V$NKX61.02 | 355 | 369 | - | 1 | 0.865 | aagaTTAAatgattt |
| P1' | V$PAX8 | PAX-2/5/8 binding sites | V$PAX8.01 | 362 | 374 | - | 0.9 | 0.9 | ctcTGAAgattaa |
| P1' | O$INRE | Core promoter initiator elements | O$DINR.01 | 379 | 389 | - | 1 | 0.945 | ttTCAGtctaa |
| P1' | V$HOMF | Homeodomain transcription factors | V$HMX2.02 | 380 | 398 | + | 1 | 0.848 | tagactgaAAACctttcaa |
| P1' | V$NRSF | Neuron-restrictive silencer factor | V$NRSF.02 | 380 | 410 | - | 1 | 0.764 | ttttcaGCACagttgaaaggttttcagtcta |
| P1' | V$HOMF | Homeodomain transcription factors | V$HMX2.02 | 385 | 403 | - | 0.75 | 0.825 | cacagttgAAAGgttttca |
| P1' | V$MYT1 | MYT1 C2HC zinc finger protein | V$MYT1L.01 | 385 | 397 | - | 0.818 | 0.927 | tgaaAGGTtttca |
| P1' | M$TFAM | Mitochondral HMG-box transcription factor | M$TFAM.01 | 405 | 417 | - | 1 | 0.92 | tctaggtTTTTca |
| P1' | V$SMAD | Vertebrate SMAD family of transcription factors | V$SMAD.01 | 418 | 426 | - | 1 | 0.964 | gGTCTgtct |
| P1' | V$KLFS | Krueppel like transcription factors | V$BKLF.01 | 426 | 444 | - | 1 | 0.95 | tgagaGGGTgggcaaaatg |
| P1' | V$HOMF | Homeodomain transcription factors | V$HMX3.02 | 436 | 454 | + | 1 | 0.952 | accctctcatTTAAaagga |
| P1' | O$VTBP | Vertebrate TATA binding protein factor | O$MTATA.01 | 437 | 453 | - | 1 | 0.854 | cctttTAAAtgagaggg |
| P1' | V$HBOX | Homeobox transcription factors | V$EN1.01 | 440 | 458 | - | 1 | 0.799 | caattcctTTTAaatgaga |
| P1' | V$SORY | SOX/SRY-sex/testis determinig and related HMG box factors | V$HMGA.01 | 440 | 456 | + | 1 | 0.887 | tctCATTtaaaaggaat |
| P1' | V$BRNF | Brn POU domain factors | V$BRN2.01 | 441 | 459 | + | 1 | 0.867 | ctCATTtaaaaggaattga |
| P1' | V$HOMF | Homeodomain transcription factors | V$HMX3.02 | 441 | 459 | - | 1 | 0.954 | tcaattccttTTAAatgag |
| P1' | V$SORY | SOX/SRY-sex/testis determinig and related HMG box factors | V$HMGA.01 | 451 | 467 | + | 1 | 0.886 | aggAATTgaagaagaaa |
| P1' | V$ABDB | Abdominal-B type homeodomain transcription factors | V$HOXA10.01 | 460 | 476 | + | 1 | 0.922 | agaagaaaTAAAatggc |
| P1' | V$CDXF | Vertebrate caudal related homeodomain protein | V$CDX2.02 | 460 | 478 | - | 1 | 0.908 | ctgccattTTATttcttct |
| P1' | V$OCT1 | Octamer binding protein | V$OCT1.05 | 460 | 476 | - | 0.9 | 0.894 | gcCATTttatttcttct |
| P1' | V$BRNF | Brn POU domain factors | V$TST1.01 | 461 | 479 | + | 0.9 | 0.941 | gaagaAATAaaatggcaga |
| P1' | V$YY1F | Activator/repressor binding to transcription initiation site | V$YY1.01 | 462 | 480 | - | 1 | 0.874 | ctctgCCATtttatttctt |
| P1' | V$RXRF | RXR heterodimer binding sites | V$RAR_RXR.01 | 471 | 495 | + | 1 | 0.808 | aatggcagaggtttaAGGTtactat |
| P1' | V$NR2F | Nuclear receptor subfamily 2 factors | V$TR4.02 | 473 | 497 | + | 1 | 0.803 | tggcagAGGTttaaggttactattc |
| P2 | V$SORY | SOX/SRY-sex/testis determinig and related HMG box factors | V$SOX2.01 | 6 | 22 | - | 1 | 0.926 | gcaagACAAagagagct |
| P2 | O$VTBP | Vertebrate TATA binding protein factor | O$VTATA.02 | 23 | 39 | - | 1 | 0.913 | gtcttTAAAattgcttt |
| P2 | V$BRNF | Brn POU domain factors | V$BRN2.01 | 26 | 44 | + | 0.933 | 0.868 | gcAATTttaaagacaaact |
| P2 | V$ABDB | Abdominal-B type homeodomain transcription factors | V$HOXC10.01 | 27 | 43 | - | 0.813 | 0.842 | gtttgtctTTAAaattg |
| P2 | V$IRFF | Interferon regulatory factors | V$ISRE.01 | 29 | 49 | + | 0.75 | 0.814 | attttaaagaCAAActgccaa |
| P2 | V$TALE | TALE homeodomain class recognizing TG motifs | V$MEIS1.02 | 37 | 53 | + | 0.755 | 0.84 | gacaaactGCCAaaaca |
| P2 | V$DMRT | DM domain-containing transcription factors | V$DMRT1.01 | 66 | 86 | + | 0.828 | 0.775 | tgctctagaaaaaCTGTtgtg |
| P2 | W$RVUP | Retroviral upstream element | W$LTRUP.01 | 69 | 89 | - | 0.761 | 0.837 | tgccacaacagTTTTtctaga |
| P2 | V$FKHD | Fork head domain factors | V$FHXB.01 | 75 | 91 | - | 0.909 | 0.907 | gttgccACAAcagtttt |
| P2 | V$NKXH | NKX homeodomain factors | V$NKX25.01 | 95 | 113 | - | 1 | 1 | tgcttcAAGTgccacaaat |
| P2 | V$HESF | Vertebrate homologues of enhancer of split complex | V$DEC2.01 | 99 | 113 | + | 0.87 | 0.965 | gtggcaCTTGaagca |
| P2 | W$LDPS | Lentiviral Poly A downstream element | W$LDSPOLYA.01 | 108 | 122 | - | 0.98 | 0.89 | gaCTGTgtctgcttc |
| P2 | V$PAX5 | PAX-5 B-cell-specific activator protein | V$PAX5.03 | 125 | 153 | + | 0.894 | 0.807 | tagtgCCACagccccgtgttttccatcga |
| P2 | V$NFAT | Nuclear factor of activated T-cells | V$NFAT5.01 | 133 | 151 | - | 1 | 0.891 | gatGGAAaacacggggctg |
| P2 | V$EBOX | E-box binding factors | V$MAX.01 | 134 | 146 | - | 1 | 0.859 | aaaaCACGgggct |
| P2 | V$FKHD | Fork head domain factors | V$ILF1.01 | 136 | 152 | - | 1 | 0.991 | cgatggaaAACAcgggg |
| P2 | V$CLOX | CLOX and CLOX homology (CDP) factors | V$CDPCR3HD.01 | 146 | 164 | - | 0.929 | 0.946 | caacatgGATGtcgatgga |
| P2 | V$FKHD | Fork head domain factors | V$XFD3.01 | 155 | 171 | - | 1 | 0.836 | gccaagtcAACAtggat |
| P2 | V$MYT1 | MYT1 C2HC zinc finger protein | V$MYT1.01 | 156 | 168 | + | 0.75 | 0.802 | tccATGTtgactt |
| P2 | V$AP1R | MAF and AP1 related factors | V$VMAF.01 | 157 | 177 | + | 1 | 0.85 | ccatgtTGACttggccaccgt |
| P2 | V$HNF6 | Onecut homeodomain factor HNF6 | V$HNF6.01 | 157 | 173 | - | 1 | 0.825 | tggccaagTCAAcatgg |
| P2 | V$HOMF | Homeodomain transcription factors | V$HMX2.02 | 169 | 187 | - | 1 | 0.86 | cagtactgAAACggtggcc |
| P2 | V$MYBL | Cellular and viral myb-like transcriptional regulators | V$VMYB.05 | 169 | 181 | - | 1 | 0.944 | tgaAACGgtggcc |
| P2 | V$ZFHX | Two-handed zinc finger homeodomain transcription factors | V$AREB6.04 | 171 | 183 | + | 1 | 0.988 | ccaccGTTTcagt |
| P2 | V$NKXH | NKX homeodomain factors | V$NKX31.02 | 174 | 192 | + | 1 | 0.825 | ccgtttcaGTACtgtgtct |
| P2 | V$BRN5 | Brn-5 POU domain factors | V$BRN5.03 | 200 | 222 | + | 0.75 | 0.789 | cTAAGgaggctgtaaagtgcttt |
| P2 | W$RVUP | Retroviral upstream element | W$LTRUP.01 | 206 | 226 | - | 0.777 | 0.822 | gacaaaagcacTTTAcagcct |
| P2 | V$NKXH | NKX homeodomain factors | V$NKX23.01 | 208 | 226 | + | 0.855 | 0.859 | gctgtaaaGTGCttttgtc |
| P2 | V$NKXH | NKX homeodomain factors | V$NKX29.01 | 209 | 227 | - | 0.839 | 0.852 | ggacaaaaGCACtttacag |
| P2 | V$FAST | FAST-1 SMAD interacting proteins | V$FAST1.01 | 218 | 234 | + | 0.85 | 0.83 | gcttttgtcCATTcctt |
| P2 | V$HOXF | "Paralog hox genes 1-8 from the four hox clusters A, B, C, D" | V$NANOG.01 | 219 | 237 | - | 1 | 0.945 | aaaaaggAATGgacaaaag |
| P2 | V$ETSF | Human and murine ETS1 factors | V$SPIB.01 | 220 | 240 | - | 1 | 0.889 | gacaaaaaGGAAtggacaaaa |
| P2 | V$KLFS | Krueppel like transcription factors | V$GKLF.01 | 220 | 238 | - | 0.779 | 0.892 | caaaaaggaATGGacaaaa |
| P2 | V$SORY | SOX/SRY-sex/testis determinig and related HMG box factors | V$HBP1.01 | 222 | 238 | - | 1 | 0.882 | caaaaaggAATGgacaa |
| P2 | V$TEAF | TEA/ATTS DNA binding domain factors | V$TEAD.01 | 224 | 236 | + | 1 | 0.924 | gtcCATTcctttt |
| P2 | V$HNF1 | Hepatic Nuclear Factor 1 | V$HNF1.04 | 239 | 255 | - | 1 | 0.854 | acccctttGTTAaaaga |
| P2 | V$LEFF | LEF1/TCF | V$LEF1.01 | 240 | 256 | + | 1 | 0.904 | cttttaaCAAAggggtt |
| P2 | V$KLFS | Krueppel like transcription factors | V$GKLF.02 | 241 | 259 | + | 1 | 0.966 | ttttaacAAAGgggtttac |
| P2 | V$SORY | SOX/SRY-sex/testis determinig and related HMG box factors | V$SOX2.01 | 241 | 257 | + | 1 | 0.984 | ttttaACAAaggggttt |
| P2 | V$BNCF | Basonuclein rDNA transcription factor (PolI) | V$BNC.01 | 282 | 300 | + | 0.842 | 0.87 | agtggcgatgTGTTcctgt |
| P2 | N$MAB3 | C.elegans homolog of Drosophila doublesex | N$MAB3.01 | 296 | 308 | + | 1 | 0.848 | cctGTTGcggatt |
| P2 | V$AP1R | MAF and AP1 related factors | V$MARE.02 | 296 | 316 | - | 1 | 0.936 | gaaTGCTtaatccgcaacagg |
| P2 | V$AP1R | MAF and AP1 related factors | V$MARE.02 | 298 | 318 | + | 0.791 | 0.881 | tgtTGCGgattaagcattcaa |
| P2 | V$BCDF | Bicoid-like homeodomain transcription factors | V$DMBX1.01 | 298 | 314 | - | 1 | 0.968 | atgcttAATCcgcaaca |
| P2 | V$HOXF | "Paralog hox genes 1-8 from the four hox clusters A, B, C, D" | V$NANOG.01 | 304 | 322 | - | 1 | 0.954 | tgatttgAATGcttaatcc |
| P2 | V$SORY | SOX/SRY-sex/testis determinig and related HMG box factors | V$HMGA.01 | 309 | 325 | + | 1 | 0.882 | aagCATTcaaatcaatg |
| P2 | V$CLOX | CLOX and CLOX homology (CDP) factors | V$CDPCR3HD.01 | 310 | 328 | - | 0.885 | 0.948 | actcattGATTtgaatgct |
| P2 | V$OCT1 | Octamer binding protein | V$OCT1.02 | 310 | 326 | + | 0.75 | 0.851 | agcATTCaaatcaatga |
| P2 | V$BRNF | Brn POU domain factors | V$BRN2.03 | 311 | 329 | - | 1 | 0.964 | gactcattgATTTgaatgc |
| P2 | V$HNF6 | Onecut homeodomain factor HNF6 | V$HNF6.01 | 312 | 328 | + | 1 | 0.947 | cattcaaaTCAAtgagt |
| P2 | V$SORY | SOX/SRY-sex/testis determinig and related HMG box factors | V$HMGA.01 | 312 | 328 | - | 1 | 0.918 | actCATTgatttgaatg |
| P2 | V$SORY | SOX/SRY-sex/testis determinig and related HMG box factors | V$HBP1.01 | 314 | 330 | + | 1 | 0.876 | ttcaaatcAATGagtct |
| P2 | V$CLOX | CLOX and CLOX homology (CDP) factors | V$CLOX.01 | 315 | 333 | + | 0.873 | 0.824 | tcaaATCAatgagtctctt |
| P2 | V$GFI1 | Growth factor independence transcriptional repressor | V$GFI1.02 | 315 | 329 | + | 1 | 0.934 | tcaAATCaatgagtc |
| P2 | V$GFI1 | Growth factor independence transcriptional repressor | V$GFI1.02 | 335 | 349 | - | 1 | 0.903 | gcaAATCaaactaag |
| P2 | V$CAAT | CCAAT binding factors | V$NFY.03 | 338 | 352 | - | 0.75 | 0.845 | ctagCAAAtcaaact |
| P2 | V$YBXF | "Y-box binding transcription factors, multifunctional proteins involved in transcriptional and translational regulation, mRNA splicing, DNA replication and repair" | V$YB1.01 | 341 | 353 | + | 0.818 | 0.883 | ttgatTTGCtagc |
| P2 | W$RPOA | Retroviral PolyA signal | W$POLYA.01 | 345 | 365 | - | 1 | 0.765 | cagatTAAAccagctagcaaa |
| P2 | V$CP2F | CP2-erythrocyte Factor related to drosophila Elf1 | V$TCFCP2L1.01 | 351 | 369 | + | 1 | 0.876 | agCTGGtttaatctgtttt |
| P2 | V$GATA | GATA binding factors | V$GATA3.02 | 355 | 367 | - | 1 | 0.927 | aacAGATtaaacc |
| P2 | V$FKHD | Fork head domain factors | V$ILF1.01 | 359 | 375 | - | 1 | 0.98 | ctacacaaAACAgatta |
| P2 | V$SF1F | Vertebrate steroidogenic factor | V$FTF.01 | 374 | 386 | - | 1 | 0.946 | gctcCAAGgcact |
| P2 | V$CREB | cAMP-responsive element binding proteins | V$TAXCREB.01 | 381 | 401 | + | 0.83 | 0.81 | tggagcTTACgcatttttttt |
| P2 | V$PAX6 | PAX-4/PAX-6 paired domain binding sites | V$PAX6.01 | 384 | 402 | + | 1 | 0.774 | agcttACGCattttttttt |
| P2 | N$CEDS | Cell-death specification 2 | N$CES2.01 | 385 | 395 | - | 1 | 0.779 | aatgcGTAAgc |
| P2 | V$E2FF | E2F-myc activator/cell cycle regulator | V$E2F.01 | 400 | 416 | - | 1 | 0.776 | gaatggaggGAAAaaaa |
| P2 | V$IRFF | Interferon regulatory factors | V$IRF7.01 | 406 | 426 | - | 0.936 | 0.881 | tagtGAATtcgaatggaggga |
| P2 | V$PAX8 | PAX-2/5/8 binding sites | V$PAX8.01 | 430 | 442 | - | 0.85 | 0.884 | cccTCATgcttgc |
| P2 | V$GCMF | Chorion-specific transcription factors with a GCM DNA binding domain | V$GCM1.01 | 434 | 444 | - | 1 | 0.957 | atCCCTcatgc |
| P2 | V$GATA | GATA binding factors | V$GATA2.02 | 438 | 450 | + | 1 | 0.906 | gaggGATAtgaga |
| P2 | V$FKHD | Fork head domain factors | V$XFD3.01 | 446 | 462 | + | 1 | 0.822 | tgagagtgAACAtggct |
| P2 | V$YY1F | Activator/repressor binding to transcription initiation site | V$YY1.01 | 447 | 465 | - | 1 | 0.858 | tgcagCCATgttcactctc |
| P2 | V$MYBL | Cellular and viral myb-like transcriptional regulators | V$VMYB.01 | 459 | 471 | - | 0.865 | 0.882 | aaaAACTgcagcc |
| P2 | V$E2FF | E2F-myc activator/cell cycle regulator | V$E2F.01 | 465 | 481 | - | 1 | 0.762 | cttaagcagGAAAaact |
| P2 | V$HOMF | Homeodomain transcription factors | V$HMX2.01 | 467 | 485 | + | 1 | 0.9 | tttttcctgCTTAagcttg |
| P2 | V$HOMF | Homeodomain transcription factors | V$HMX2.01 | 472 | 490 | - | 1 | 0.92 | caaagcaagCTTAagcagg |
| P2 | V$LEFF | LEF1/TCF | V$LEF1.02 | 481 | 497 | - | 1 | 0.967 | aaaggatCAAAgcaagc |
| P3 | V$MYT1 | MYT1 C2HC zinc finger protein | V$MYT1.01 | 1 | 13 | + | 0.75 | 0.9 | acaCAGTttactt |
| P3 | V$MYT1 | MYT1 C2HC zinc finger protein | V$MYT1.01 | 4 | 16 | - | 1 | 0.775 | tgaAAGTaaactg |
| P3 | V$PAX2 | PAX-2 binding sites | V$PAX2.01 | 4 | 26 | - | 1 | 0.804 | taggatttcctgaaagtAAACtg |
| P3 | V$STAT | Signal transducer and activator of transcription | V$STAT3.01 | 6 | 24 | - | 1 | 0.841 | ggatTTCCtgaaagtaaac |
| P3 | V$STAT | Signal transducer and activator of transcription | V$STAT.01 | 8 | 26 | + | 1 | 0.911 | ttactttcaGGAAatccta |
| P3 | V$ETSF | Human and murine ETS1 factors | V$ETS2.01 | 9 | 29 | + | 1 | 0.881 | tactttcAGGAaatcctaatt |
| P3 | M$TFAM | Mitochondral HMG-box transcription factor | M$TFAM.01 | 16 | 28 | - | 0.916 | 0.942 | attaggaTTTCct |
| P3 | V$HBOX | Homeobox transcription factors | V$EN1.02 | 18 | 36 | - | 1 | 0.866 | agaaaaaAATTaggatttc |
| P3 | V$HOMF | Homeodomain transcription factors | V$MSX2.01 | 18 | 36 | + | 1 | 0.964 | gaaatcCTAAtttttttct |
| P3 | V$OCT1 | Octamer binding protein | V$OCT1.06 | 18 | 34 | + | 1 | 0.849 | gaaatcctAATTttttt |
| P3 | V$BRN5 | Brn-5 POU domain factors | V$BRN5.03 | 20 | 42 | - | 1 | 0.852 | aTAATtagaaaaaaattaggatt |
| P3 | V$NKXH | NKX homeodomain factors | V$NKX25.02 | 20 | 38 | + | 1 | 0.895 | aatccTAATttttttctaa |
| P3 | V$CART | Cart-1 (cartilage homeoprotein 1) | V$S8.01 | 26 | 46 | - | 1 | 0.997 | tttcaTAATtagaaaaaaatt |
| P3 | V$ATBF | AT-binding transcription factor | V$ATBF1.01 | 27 | 43 | + | 1 | 0.888 | atttttttctAATTatg |
| P3 | V$BRN5 | Brn-5 POU domain factors | V$BRN5.04 | 27 | 49 | + | 1 | 0.909 | atttttttctaATTAtgaaagaa |
| P3 | V$DLXF | Distal-less homeodomain transcription factors | V$DLX1.01 | 27 | 45 | + | 1 | 0.982 | atttttttctAATTatgaa |
| P3 | V$LHXF | Lim homeodomain factors | V$LHX6.01 | 27 | 49 | + | 1 | 0.892 | atttttttcTAATtatgaaagaa |
| P3 | V$PDX1 | Pancreatic and intestinal homeodomain transcription factor | V$IPF1.01 | 27 | 45 | + | 1 | 0.906 | atttttttcTAATtatgaa |
| P3 | V$HOXF | "Paralog hox genes 1-8 from the four hox clusters A, B, C, D" | V$HOXC8.01 | 28 | 46 | - | 1 | 0.948 | tttcataATTAgaaaaaaa |
| P3 | V$LHXF | Lim homeodomain factors | V$LHX6.01 | 28 | 50 | - | 1 | 0.887 | tttctttcaTAATtagaaaaaaa |
| P3 | V$NKXH | NKX homeodomain factors | V$NKX25.02 | 28 | 46 | - | 1 | 0.95 | tttcaTAATtagaaaaaaa |
| P3 | V$BRNF | Brn POU domain factors | V$BRN2.04 | 29 | 47 | + | 1 | 0.869 | ttttttcTAATtatgaaag |
| P3 | V$HBOX | Homeobox transcription factors | V$GSH2.01 | 29 | 47 | - | 1 | 0.955 | ctttcaTAATtagaaaaaa |
| P3 | V$HOMF | Homeodomain transcription factors | V$MSX.01 | 29 | 47 | + | 1 | 0.983 | ttttttcTAATtatgaaag |
| P3 | V$BRNF | Brn POU domain factors | V$BRN2.04 | 30 | 48 | - | 1 | 0.876 | tctttcaTAATtagaaaaa |
| P3 | V$HBOX | Homeobox transcription factors | V$GSH2.01 | 30 | 48 | + | 1 | 0.952 | tttttcTAATtatgaaaga |
| P3 | V$HOMF | Homeodomain transcription factors | V$NOBOX.01 | 30 | 48 | - | 1 | 0.936 | tctttcaTAATtagaaaaa |
| P3 | V$OCT1 | Octamer binding protein | V$OCT1.03 | 30 | 46 | + | 1 | 0.94 | tttttctaATTAtgaaa |
| P3 | V$CART | Cart-1 (cartilage homeoprotein 1) | V$S8.01 | 31 | 51 | + | 1 | 0.992 | ttttcTAATtatgaaagaaac |
| P3 | V$HOXF | "Paralog hox genes 1-8 from the four hox clusters A, B, C, D" | V$HOXA3.01 | 31 | 49 | + | 1 | 0.943 | ttttcTAATtatgaaagaa |
| P3 | V$OCT1 | Octamer binding protein | V$OCT1.03 | 31 | 47 | - | 1 | 0.942 | ctttcataATTAgaaaa |
| P3 | V$PAXH | PAX homeodomain binding sites | V$PAX4.02 | 31 | 45 | - | 1 | 0.904 | ttcatAATTagaaaa |
| P3 | V$DLXF | Distal-less homeodomain transcription factors | V$DLX1.01 | 32 | 50 | - | 1 | 0.976 | tttctttcatAATTagaaa |
| P3 | V$PAXH | PAX homeodomain binding sites | V$PAX4.02 | 32 | 46 | + | 1 | 0.907 | tttctAATTatgaaa |
| P3 | V$PDX1 | Pancreatic and intestinal homeodomain transcription factor | V$IPF1.01 | 32 | 50 | - | 1 | 0.923 | tttctttcaTAATtagaaa |
| P3 | V$ATBF | AT-binding transcription factor | V$ATBF1.01 | 34 | 50 | - | 1 | 0.852 | tttctttcatAATTaga |
| P3 | V$PARF | PAR/bZIP family | V$DBP.01 | 34 | 50 | + | 1 | 0.886 | tctaaTTATgaaagaaa |
| P3 | V$CEBP | Ccaat/Enhancer Binding Protein | V$CEBPB.01 | 35 | 49 | + | 1 | 0.946 | ctaattatGAAAgaa |
| P3 | N$CEDS | Cell-death specification 2 | N$CES2.01 | 38 | 48 | + | 0.798 | 0.772 | attatGAAAga |
| P3 | V$FKHD | Fork head domain factors | V$HFH2.01 | 41 | 57 | + | 1 | 0.962 | atgaaagaAACAttagg |
| P3 | V$LHXF | Lim homeodomain factors | V$ISL1.01 | 42 | 64 | - | 1 | 0.901 | tcaagtgccTAATgtttctttca |
| P3 | V$ZFHX | Two-handed zinc finger homeodomain transcription factors | V$AREB6.04 | 44 | 56 | - | 1 | 0.983 | ctaatGTTTcttt |
| P3 | V$BRNF | Brn POU domain factors | V$BRN4.01 | 48 | 66 | - | 1 | 0.909 | cttcaagtgccTAATgttt |
| P3 | V$NKXH | NKX homeodomain factors | V$NKX23.01 | 49 | 67 | + | 0.855 | 0.823 | aacattagGCACttgaagt |
| P3 | V$NKXH | NKX homeodomain factors | V$NKX25.01 | 50 | 68 | - | 1 | 1 | tacttcAAGTgcctaatgt |
| P3 | V$HESF | Vertebrate homologues of enhancer of split complex | V$DEC2.01 | 54 | 68 | + | 0.87 | 0.965 | taggcaCTTGaagta |
| P3 | V$HBOX | Homeobox transcription factors | V$EN1.01 | 55 | 73 | - | 0.782 | 0.771 | ttaactacTTCAagtgcct |
| P3 | V$LHXF | Lim homeodomain factors | V$LHX3.02 | 58 | 80 | + | 1 | 0.852 | cacttgaagtagtTAATttcccc |
| P3 | V$LHXF | Lim homeodomain factors | V$LHX3.01 | 59 | 81 | - | 1 | 0.865 | aggggaaaTTAActacttcaagt |
| P3 | V$BRNF | Brn POU domain factors | V$BRN4.01 | 60 | 78 | + | 1 | 0.913 | cttgaagtagtTAATttcc |
| P3 | V$BRNF | Brn POU domain factors | V$BRN4.02 | 61 | 79 | - | 1 | 0.851 | gggaaaTTAActacttcaa |
| P3 | V$HNF1 | Hepatic Nuclear Factor 1 | V$HMBOX.01 | 61 | 77 | + | 1 | 0.892 | ttgaagtaGTTAatttc |
| P3 | V$DLXF | Distal-less homeodomain transcription factors | V$DLX5.01 | 62 | 80 | + | 1 | 0.924 | tgaagtagttAATTtcccc |
| P3 | V$LHXF | Lim homeodomain factors | V$LHX3.01 | 62 | 84 | + | 1 | 0.869 | tgaagtagTTAAtttcccctagc |
| P3 | V$PDX1 | Pancreatic and intestinal homeodomain transcription factor | V$PDX1.01 | 62 | 80 | + | 1 | 0.75 | tgaagtagtTAATttcccc |
| P3 | V$HOXF | "Paralog hox genes 1-8 from the four hox clusters A, B, C, D" | V$HOXB8.01 | 63 | 81 | - | 1 | 0.875 | aggggaaATTAactacttc |
| P3 | V$SORY | SOX/SRY-sex/testis determinig and related HMG box factors | V$HMGIY.01 | 63 | 79 | - | 1 | 0.921 | gggaAATTaactacttc |
| P3 | O$VTBP | Vertebrate TATA binding protein factor | O$ATATA.01 | 64 | 80 | + | 0.75 | 0.797 | aagtagtTAATttcccc |
| P3 | V$BRNF | Brn POU domain factors | V$BRN4.02 | 64 | 82 | + | 1 | 0.804 | aagtagTTAAtttccccta |
| P3 | V$HBOX | Homeobox transcription factors | V$VAX1.01 | 64 | 82 | - | 1 | 0.844 | taggggaAATTaactactt |
| P3 | V$PIT1 | GHF-1 pituitary specific pou domain transcription factor | V$PIT1.02 | 64 | 78 | - | 1 | 0.841 | ggaaaTTAActactt |
| P3 | V$ABDB | Abdominal-B type homeodomain transcription factors | V$HOXC9.01 | 65 | 81 | - | 1 | 0.871 | aggggaaaTTAActact |
| P3 | V$PIT1 | GHF-1 pituitary specific pou domain transcription factor | V$PIT1.02 | 65 | 79 | + | 1 | 0.816 | agtagTTAAtttccc |
| P3 | V$HOXF | "Paralog hox genes 1-8 from the four hox clusters A, B, C, D" | V$HOXA3.01 | 66 | 84 | + | 1 | 0.865 | gtagtTAATttcccctagc |
| P3 | V$NKX6 | NK6 homeobox transcription factors | V$NKX61.01 | 66 | 80 | + | 1 | 0.96 | gtagTTAAtttcccc |
| P3 | V$NKXH | NKX homeodomain factors | V$NKX25.02 | 66 | 84 | + | 1 | 0.884 | gtagtTAATttcccctagc |
| P3 | V$DLXF | Distal-less homeodomain transcription factors | V$DLX1.02 | 67 | 85 | - | 1 | 0.88 | cgctaggggaAATTaacta |
| P3 | V$MZF1 | Myeloid zinc finger 1 factors | V$MZF1.02 | 72 | 82 | - | 1 | 1 | taGGGGaaatt |
| P3 | V$HEAT | Heat shock factors | V$HSF1.03 | 81 | 105 | - | 0.868 | 0.834 | cctgtaaccactTGAAagatcgcta |
| P3 | V$MYT1 | MYT1 C2HC zinc finger protein | V$MYT1L.01 | 81 | 93 | - | 0.818 | 0.927 | tgaaAGATcgcta |
| P3 | V$HOMF | Homeodomain transcription factors | V$HMX3.01 | 85 | 103 | + | 1 | 0.911 | gatctttcAAGTggttaca |
| P3 | V$NKXH | NKX homeodomain factors | V$NKX25.01 | 87 | 105 | + | 1 | 1 | tctttcAAGTggttacagg |
| P3 | V$DMRT | DM domain-containing transcription factors | V$DMRT3.01 | 91 | 111 | + | 1 | 0.824 | tcaagtggtTACAggatctgt |
| P3 | V$PARF | PAR/bZIP family | V$VBP.01 | 95 | 111 | - | 1 | 0.872 | acagatcctGTAAccac |
| P3 | V$DMRT | DM domain-containing transcription factors | V$DMRT5.01 | 103 | 123 | + | 0.808 | 0.818 | aggatctGTGAcattattggt |
| P3 | V$CREB | cAMP-responsive element binding proteins | V$ATF.02 | 105 | 125 | + | 1 | 0.832 | gatctgTGACattattggttg |
| P3 | V$HOXF | "Paralog hox genes 1-8 from the four hox clusters A, B, C, D" | V$HOX1-3.01 | 105 | 123 | - | 1 | 0.822 | accaaTAATgtcacagatc |
| P3 | V$E4FF | Ubiquitous GLI - Krueppel like zinc finger involved in cell cycle regulation | V$E4F.01 | 107 | 119 | - | 0.842 | 0.83 | ataATGTcacaga |
| P3 | V$CLOX | CLOX and CLOX homology (CDP) factors | V$CDP.02 | 109 | 127 | - | 1 | 0.95 | aacaacCAATaatgtcaca |
| P3 | V$HBOX | Homeobox transcription factors | V$GSH1.01 | 110 | 128 | - | 1 | 0.857 | gaacaaccaaTAATgtcac |
| P3 | V$CAAT | CCAAT binding factors | V$CAAT.01 | 112 | 126 | - | 1 | 0.941 | acaaCCAAtaatgtc |
| P3 | V$GREF | Glucocorticoid responsive and related elements | V$PRE.01 | 113 | 131 | + | 1 | 0.902 | acattattggtTGTTctgt |
| P3 | V$CREB | cAMP-responsive element binding proteins | V$E4BP4.01 | 120 | 140 | + | 1 | 0.835 | tggttgttctGTAAagctcct |
| P3 | V$PARF | PAR/bZIP family | V$TEF.01 | 121 | 137 | + | 1 | 0.887 | ggttgttctGTAAagct |
| P3 | V$PARF | PAR/bZIP family | V$TEF_HLF.01 | 122 | 138 | - | 1 | 0.801 | gagctTTACagaacaac |
| P3 | W$RPAD | Retroviral PolyA Downstream signal | W$PADS.01 | 139 | 151 | - | 1 | 0.871 | tggGTGGtctcag |
| P3 | V$GLIF | GLI zinc finger family | V$GLI3.01 | 141 | 155 | + | 1 | 0.973 | gagaCCACccacttt |
| P3 | V$NKXH | NKX homeodomain factors | V$NKX26.01 | 142 | 160 | - | 1 | 0.838 | aggagaaAGTGggtggtct |
| P3 | V$HOMF | Homeodomain transcription factors | V$HMX3.01 | 144 | 162 | - | 1 | 0.931 | ggaggagaAAGTgggtggt |
| P3 | V$PRDF | Positive regulatory domain I binding factor | V$BLIMP1.01 | 144 | 162 | - | 1 | 0.871 | ggaggaGAAAgtgggtggt |
| P3 | V$RXRF | RXR heterodimer binding sites | V$VDR_RXR.06 | 151 | 175 | - | 0.937 | 0.776 | gagatggtaaaggggAGGAgaaagt |
| P3 | V$MAZF | Myc associated zinc fingers | V$MAZ.01 | 153 | 165 | - | 1 | 0.939 | agggGAGGagaaa |
| P3 | V$MZF1 | Myeloid zinc finger 1 factors | V$MZF1.02 | 156 | 166 | - | 1 | 0.99 | aaGGGGaggag |
| P3 | V$ABDB | Abdominal-B type homeodomain transcription factors | V$HOXB9.01 | 160 | 176 | - | 1 | 0.9 | cgagatggTAAAgggga |
| P3 | V$NRSF | Neuron-restrictive silencer factor | V$NRSE.01 | 163 | 193 | + | 1 | 0.696 | cctttaccatctCGGAtgaaaaccataacaa |
| P3 | V$OCT1 | Octamer binding protein | V$OCT.01 | 175 | 191 | + | 0.857 | 0.8 | cggATGAaaaccataac |
| P3 | V$FKHD | Fork head domain factors | V$FHXB.01 | 181 | 197 | + | 1 | 0.86 | aaaaccATAAcaactcc |
| P3 | V$OCT1 | Octamer binding protein | V$OCT3_4.02 | 196 | 212 | - | 1 | 0.912 | actgtGCATgcttgtgg |
| P3 | V$OCT1 | Octamer binding protein | V$OCT1.05 | 204 | 220 | - | 0.95 | 0.89 | caCATCacactgtgcat |
| P3 | B$CRBS | CRP binding site | B$CRP.01 | 210 | 230 | + | 1 | 0.915 | agTGTGatgtggttcagaggc |
| P3 | V$HAML | Human acute myelogenous leukemia factors | V$AML3.01 | 214 | 228 | + | 1 | 0.863 | tgatGTGGttcagag |
| P3 | V$STAT | Signal transducer and activator of transcription | V$STAT6.01 | 217 | 235 | - | 0.758 | 0.843 | aagcTGCCtctgaaccaca |
| P3 | V$STAT | Signal transducer and activator of transcription | V$STAT6.01 | 256 | 274 | - | 1 | 0.91 | gctcTTCCtctgaatacaa |
| P3 | V$STAT | Signal transducer and activator of transcription | V$STAT6.01 | 257 | 275 | + | 0.862 | 0.887 | tgtaTTCAgaggaagagca |
| P3 | V$ETSF | Human and murine ETS1 factors | V$PEA3.01 | 259 | 279 | + | 1 | 0.942 | tattcagAGGAagagcatcat |
| P3 | N$MAB3 | C.elegans homolog of Drosophila doublesex | N$MAB3.01 | 279 | 291 | - | 1 | 0.895 | gatGTTGccaaaa |
| P3 | V$HAND | Twist subfamily of class B bHLH transcription factors | V$TAL1ALPHAE47.01 | 280 | 300 | - | 1 | 0.909 | tcactttCAGAtgttgccaaa |
| P3 | V$IRFF | Interferon regulatory factors | V$IRF2.01 | 289 | 309 | + | 1 | 0.964 | atctgaaagtGAAAacggaag |
| P3 | V$PRDF | Positive regulatory domain I binding factor | V$BLIMP1.01 | 293 | 311 | + | 1 | 0.909 | gaaagtGAAAacggaagcc |
| P3 | V$IRFF | Interferon regulatory factors | V$IRF3.01 | 295 | 315 | + | 0.758 | 0.87 | aagtgaaaacGGAAgccagaa |
| P3 | V$NF1F | Nuclear factor 1 | V$NF1.03 | 296 | 316 | + | 1 | 0.924 | agtgaaaacggaaGCCAgaaa |
| P3 | V$ETSF | Human and murine ETS1 factors | V$NRF2.01 | 297 | 317 | + | 1 | 0.93 | gtgaaaacGGAAgccagaaac |
| P3 | V$MYBL | Cellular and viral myb-like transcriptional regulators | V$VMYB.05 | 299 | 311 | + | 1 | 0.942 | gaaAACGgaagcc |
| P3 | V$HAND | Twist subfamily of class B bHLH transcription factors | V$TH1E47.01 | 301 | 321 | + | 1 | 0.935 | aaacggaagCCAGaaacactt |
| P3 | V$ZFHX | Two-handed zinc finger homeodomain transcription factors | V$AREB6.04 | 310 | 322 | - | 1 | 0.981 | caagtGTTTctgg |
| P3 | V$P53F | p53 tumor suppressor | V$P53.01 | 314 | 336 | + | 0.844 | 0.734 | aaacaCTTGgccagccctggggg |
| P3 | V$NOLF | Neuron-specific-olfactory factor | V$OLF1.02 | 321 | 343 | - | 1 | 0.905 | aaaaaaTCCCccagggctggcca |
| P3 | V$TEAF | TEA/ATTS DNA binding domain factors | V$TEAD.01 | 359 | 371 | - | 1 | 0.943 | tgtCATTccacca |
| P3 | O$INRE | Core promoter initiator elements | O$DINR.01 | 361 | 371 | - | 0.968 | 0.949 | tgTCATtccac |
| P3 | N$CSKN | Caenorhabditis maternal gene product SKN-1 | N$SKN1.01 | 362 | 374 | - | 1 | 0.969 | aaatGTCAttcca |
| P3 | V$AP1R | MAF and AP1 related factors | V$MARE.02 | 369 | 389 | - | 0.875 | 0.916 | agaTGCCtacacagcaaatgt |
| P3 | V$AP1R | MAF and AP1 related factors | V$MAFA.01 | 371 | 391 | + | 1 | 0.962 | attTGCTgtgtaggcatcttt |
| P3 | V$NFAT | Nuclear factor of activated T-cells | V$NFAT.01 | 378 | 396 | - | 1 | 0.988 | agaGGAAagatgcctacac |
| P3 | V$PLZF | C2H2 zinc finger protein PLZF | V$PLZF.01 | 392 | 406 | - | 1 | 0.918 | aaaTACAgtcagagg |
| P3 | V$EREF | Estrogen response elements | V$ERR.01 | 404 | 422 | - | 1 | 0.907 | actcttcAAGGccaagaaa |
| P3 | V$SF1F | Vertebrate steroidogenic factor | V$SF1.01 | 408 | 420 | - | 1 | 1 | tcttCAAGgccaa |
| P3 | V$NKXH | NKX homeodomain factors | V$NKX31.02 | 414 | 432 | - | 1 | 0.811 | taaactcaGTACtcttcaa |
| P3 | V$PAX2 | PAX-2 binding sites | V$PAX2.01 | 415 | 437 | + | 0.789 | 0.811 | tgaagagtactgagtttAAAAag |
| P3 | V$BRN5 | Brn-5 POU domain factors | V$BRN5.03 | 421 | 443 | + | 0.75 | 0.765 | gTACTgagtttaaaaagacagta |
| P3 | B$LEXA | Bacterial LexA repressor protein involved in SOS response | B$LEXA.01 | 424 | 444 | - | 1 | 0.713 | ataCTGTctttttaaactcag |
| P3 | V$TALE | TALE homeodomain class recognizing TG motifs | V$MEIS1.01 | 441 | 457 | - | 1 | 0.954 | catggacTGTCacatac |
| P3 | V$OCT1 | Octamer binding protein | V$OCT1.06 | 452 | 468 | + | 1 | 0.901 | tccatggaAATTgcctc |
| P3 | V$NFKB | Nuclear factor kappa B/c-rel | V$HIVEP1.01 | 456 | 468 | - | 0.75 | 0.843 | gaGGCAatttcca |
| P3 | V$STAT | Signal transducer and activator of transcription | V$STAT5.01 | 463 | 481 | - | 0.845 | 0.946 | agatTTCAcagaagaggca |
| P3 | V$STAT | Signal transducer and activator of transcription | V$STAT5.01 | 465 | 483 | + | 1 | 0.945 | cctcTTCTgtgaaatctcg |
| P3 | V$MYOD | Myoblast determining factors | V$E47.01 | 480 | 496 | - | 1 | 0.95 | tcggaGCAGgtggcgag |
| P3 | V$NEUR | "NeuroD, Beta2, HLH domain" | V$NEUROD1.01 | 481 | 493 | + | 0.767 | 0.883 | tcgcCACCtgctc |

The transcription elements were predicted using MatInspector Professional (<http://genomatrix.de/cgi-bin/matinspector_prof/mat_fam.pl>) with default settings. The promoter sequences of 1500 bp, 500 bp, 500 bp, and 500 bp, corresponding to promoter P1 (5504534-5503035 of NT_030737), P1’ (5458744-5458245), P2 (5424838-5424339), and P3 (5400314-5399815) were used for the analysis.
