# Supplementary material for: Genomic characterization of the human mitochondrial tumor suppressor gene 1 (MTUS1): 5' cloning and preliminary analysis of the multiple gene promoters
Source: BMC Res Notes. 2009 Jun 19;2:109. doi: 10.1186/1756-0500-2-109 (PMC2706840; doi:10.1186/1756-0500-2-109)
Supplement: Additional file 3 — Table S3. Primers used to clone promoter regions of the MTUS1 gene. [file 1756-0500-2-109-S3.doc]

**Supplement Table 3**

**Primers used to clone promoter regions of the *MTUS1* gene**

| Promoter | Primer | Sequence | Product size | Genomic location  (on NT_030737) |
| --- | --- | --- | --- | --- |
| P1 | forward | 5’-ctgaggtaccggatggaggcttgttgctaa-3’ | 2286 bp | start 5505262  end 5502978 |
|  | reverse | 5’-tgtcctcgagcgttcctcagtgaacaccta-3’ |  |  |
| P1’ | forward | 5’-ctgaggtaccgggaacagttt agtgcataac-3’ | 773 bp | start 5459076  end 5458304 |
|  | reverse | 5’-tgtcctcgaggagggtgggcaaaatggtct-3’ |  |  |
| P2 | forward | 5’-cagtggtaccgaagcagacacagtctgtagt-3’ | 529 bp | start 5424731  end 5424203 |
|  | reverse | 5’-gcttctcgagccacgaccagcagtgtcaat-3’ |  |  |
| P3 | forward | 5’-ctgaggtacctgtgaggtcctcagcatgtg-3’ | 733 bp | start 5400556  end 5399824 |
|  | reverse | 5’-tgtcctcgaggcaggtggcgagatttcaca-3’ |  |  |
